# High-Resolution 4C Reveals Rapid p53-Dependent Chromatin Reorganization of the *CDKN1A* Locus in Response to Stress

**DOI:** 10.1371/journal.pone.0163885

**Published:** 2016-10-14

**Authors:** Jean-François Millau, Patrick Wijchers, Luc Gaudreau

**Affiliations:** 1 Départment de Biologie, Faculté des Sciences, Université de Sherbrooke, Sherbrooke, QC J1K 2R1, Canada; 2 Hubrecht Institute-KNAW and University Medical Center Utrecht, Uppsalalaan 8, 3584 CT Utrecht, the Netherlands; Università degli Studi di Milano, ITALY

## Abstract

A regulatory program involving hundreds of genes is coordinated by p53 to prevent carcinogenesis in response to stress. Given the importance of chromatin loops in gene regulation, we investigated whether DNA interactions participate in the p53 stress response. To shed light on this issue, we measured the binding dynamics of cohesin in response to stress. We reveal that cohesin is remodeled at specific loci during the stress response and that its binding within genes negatively correlates with transcription. At p53 target genes, stress-induced eviction of cohesin from gene bodies is concomitant to spatial reorganization of loci through the disruption of functional chromatin loops. These findings demonstrate that chromatin loops can be remodeled upon stress and contribute to the p53-driven stress response. Additionally, we also propose a mechanism whereby transcription-coupled eviction of cohesin from *CDKN1A* might act as a molecular switch to control spatial interactions between regulatory elements.

## Introduction

The cellular response to stress is a crucial mechanism that allows cells to quickly respond to aggressions coming from its environment. It requires the establishment of an intricate gene expression program coordinated by the tumor suppressor transcription factor, p53. In response to stress, p53 integrates signals and directs cell fate by regulating the expression of genes involved in major cellular pathways *e*.*g*. cell cycle control, autophagy, apoptosis, DNA repair, carbon metabolism, and reactive oxygen species detoxification [1, 2]. Depending on the type of stress and cellular context, the p53 transcriptional program leads either to cell cycle arrest, senescence, or apoptosis in order to maintain genome integrity and cellular homeostasis [3]. Gene regulation elicited by p53 in response to stress is thus important as it is one of the first barriers against carcinogenesis.

Pioneering work recently revealed that the human genome has a spatial organization shaped by chromatin loops [4–8]. The formation of these chromatin loops can require the multi-protein complex cohesin (composed of Rad21, SA1, SA2, Smc1A, and Smc3), which binds to thousands of well-conserved sites within the genome of human cells [9–11]. Using chromosome conformation capture techniques, several groups clearly demonstrated that chromatin loops are formed at cohesin binding sites and it is proposed that cohesin maintains these interactions via its unique ring-shaped structure capable of embracing two chromatin fibers [9, 11–14]. One important role of chromatin loops is to physically and functionally connect regulatory elements (*e*.*g*. enhancers, silencers) to their target promoters [4, 5, 15]. This function appears to be pivotal for gene regulation because disruption of chromatin loops, by cohesin depletion or removal of cohesin binding sites, abrogates promoter-enhancer interactions and has profound effects on target gene expression levels [16–18]. Consequently, the dynamic control of chromatin loop formation (or disruption) appears to be an important mechanism for the regulation of transcriptional pathways.

Given the importance of the spatial organization of the genome in the regulation of transcription, we explored whether DNA interactions are remodeled in response to stress to help establish the p53 transcriptional stress response program. To shed light on this issue, we used a genome-wide approach to investigate the binding of the cohesin subunit Rad21 in response to stress. Here, we show that cohesin is remodeled at specific loci during stress response. Notably, we unveil that cohesin is evicted from p53 target gene bodies during stress-induced transcription. Furthermore, using high-resolution Circular Chromosome Conformation Capture coupled to deep sequencing (4C-seq), we reveal that cohesin’s eviction from p53 target gene bodies in response to stress coincides with the disruption of chromatin loops that shape the spatial architecture of these loci. Finally, we show that these stress-dependent chromatin loops are important because they contribute to gene regulation of *CDKN1A*. Together, these findings demonstrate that chromatin loops are remodeled during the cellular stress response and contribute to the p53 transcriptional program.

## Materials and Methods

### Chemicals and reagents

Daunorubicin and Polyethylenimine (PEI) were obtained from Sigma.

### Cells, cell culture, and cell treatment

HCT116 p53+/+ and HCT116 p53-/- cells (a gift from Dr. Bert Vogelstein, Howard Hughes Medical Institute, Baltimore, MD) were cultured in a 5% CO_2_-containing atmosphere in DMEM (Wisent) supplemented with 10% fetal bovine serum (Sigma), 0.2U/mL penicillin G, and 100 mg/mL streptomycin (Invitrogen). Unless specified otherwise, cells stress treatment consisted of 250 nM daunorubicin (Sigma) for 8 h. All results presented with SEM were obtained from independent biological triplicates. Statistics were calculated using Student’s *t*-test.

### ChIP assays

ChIP assays were performed as described previously using the antibodies listed in S1 Table [19]. The recovered DNA was analyzed by qPCR using the primer sets listed in S2 Table.

### ChIP-seq assays

Rad21 ChIP-seq was performed similarly to regular ChIP experiments except for the following steps. For each treatment conditions six immuno-precipitations were performed. Immuno-precipitations were performed using Dynabeads coupled to protein A (Life Technologies) without pre-clearing. The libraries for deep-sequencing were prepared from 10 ng ChIPed DNA using the NEBNext Ultra DNA Library Prep Kit for Illumina (NEB) according to manufacturer’s protocol. Samples were sequenced paired-end at 50-bp read length on an Illumina HiSeq-2000. See supplementary S1 File for biofinformatic procedures.

### RT-qPCR

mRNAs of p21, p21C and 36B4 were quantified by RT-qPCR and normalized to 36B4 expression level. Total RNA was extracted from cultured cells using GenElute (Sigma) and reverse transcribed using the M-MLV reverse transcriptase enzyme (Enzymatics) and random hexamers according to manufacturer’s protocols. The RT-qPCR primer sets are reported in S3 Table.

### RNA-seq

Total RNA was extracted from HCT116 p53+/+ cells using GenElute (Sigma) and treated with DNaseI (Ambion). The libraries for deep-sequencing were prepared from 800 ng total RNA using Illumina’s TruSeq Stranded mRNA LT Kit according to manufacturer’s protocol. Samples were sequenced paired-end at 50-bp read length on an Illumina HiSeq-2000. See supplementary S1 File for biofinformatic procedures.

### FAIRE assay

Cells were grown in a 150 mm petri dish to 50–70% confluence and were then crosslinked in 1.1% formaldehyde for 10 min then washed two times in cold PBS. Cell pellet was resuspended in 500 μl of lysis buffer (1% SDS, 10 mM EDTA, 50 mM Tris-HCl pH 8.1, 1 mM PMSF, and 1X Roche Complete protease inhibitor cocktail) and incubated for 30 min at 4°C on a rotary wheel. Subsequently, lysate was sonicated to obtain DNA fragments of 500 bp and was then centrifugated at 14,000 rpm for 10 min at 4°C. Supernatant was recovered and a 3 μl aliquote was collected as 1% input. Next, 500 μl of phenol/chloroform and 200 μl of lysis solution were added to 300 μl of lysate, the solution was then vortexed and centifugated at 12,000 rpm for 5 min. The aqueous phase was collected and transferred to a Gel-Lock tube containing 500 μl of phenol/chloroform. The solution was then mixed by inversion and centrifugated at 14,000 rpm for 1 min. The aqueous phase was collected, transferred to a tube containing 100 μg of proteinase K, and then incubated over-night at 65°C. Parallel to this, 500 μl of TE as well as 100 μg of proteinase K were added to the input aliquote, which was then incubated over-night at 65°C. The next day, 50 μg of RNase A were added to the samples, which were then incubated for 1 h at 37°C. DNA was then purified using QIAquick PCR Purification Kit and eluted using 700 μl of elution buffer. qPCR were subsequently performed using the primers reported in the S4 Table.

### High-resolution 4C assay

4C experiments were performed and analyzed as previously described [20, 21]. Restriction enzymes used as first cutters were DpnII (New England Biolabs) or Csp61 (Fermentas), the restriction enzyme used as second cutter was BfaI (New England Biolabs). The list of 4C primers is reported in S5 Table.

### Luciferase reporter assay

We used pGL3 basic, pGL3 promoter, and pRL-SV40 (Promega) to assess promoter, enhancer and repressor activity of putative regulatory elements. Cells were cultured in six-well plates, transfected using PEI with 100 ng of pGL3 vector, 2.5 ng of pRL-SV40, and 1000 ng of pGEX as carrier DNA, and collected 24h later. When a daunorubicin treatment was performed, HCT116 cells were transfected and treated 16 h later with 250 nM daunorubicin for 8h. Firefly and Renilla luciferase activities were measured using the Dual Luciferase Reporter Assay System kit from Promega and a Lumat LB 9507 luminometer from Berthold Technologies. Firefly signal was normalized to renilla signal. The sequences cloned in pGL3 vectors are reported in the S6 Table.

### 5’RACE

5’RACE was carried out as follows. Total mRNA from HCT116 p53 +/+ cells treated with 250 nM daunorubicin for 8 h was extracted using GenElute (Sigma) and treated with DNaseI (Ambion). 1μg of total mRNA was then treated for 1 h with Tobacco Acid Pyrophosphatase (Epicentre) according to manufacturer's instructions and then purified using RNA Clean & Concentrator-5 (Zymo Research Corporation). The 5’ adaptor 5’-ACACGACGrCrUrCrUrUrCrCrGrArUrCrU-3’ was then ligated to the mRNA. A 19 μl reaction containing 1 μg of purified RNA, 40 pmoles of 5’ adaptor, 1X T4 RNA ligase 1 buffer (New England Biolabs), 10% DMSO was assembled, incubated for 1 min at 65°C and then placed on ice. The reaction was then completed by adding 1 mM ATP, 20 u of RNasin (Promega), 30 u of T4 RNA ligase 1 (New England Biolabs) for a final volume of 25 μl and was incubated for 1 h at 37°C. RNA was then purified using SPRI agencourt RNAclean XP according to manufacturer’s instructions using a volume ratio of 1.8. A reverse transcription was then performed at 45°C using M-MLV reverse transcriptase enzyme (Enzymatics) according to manufacturer’s instructions using a gene specific primer 1 (GSP1) (see S7 Table). A PCR was then performed on 2 μl of cDNA using the VerSeq polymerase (Enzymatics) according to manufacturer’s instructions with 5’-ACACGACGCTCTTCCGATCT-3’ as forward primer and GSP2 primer as reverse primer (see S7 Table). PCR products were purified using SPRI agencourt DNAclean XP according to manufacturer’s instructions using a 1.1 volume ratio. A nested PCR was subsequently performed as described above on 2 μl of PCR product using GSP3 as reverse primer. Samples were then separated on a 2% agarose gel, purified using the QIAquick Gel Extraction Kit (Qiagen), and sequenced.

## Results

### Rad21 binding negatively correlates with transcription and is evicted from the body of p53 target genes in response to stress

In order to identify loci that bear chromatin loops susceptible to be remodeled during the p53 stress response program, we investigated the genome-wide binding dynamics of cohesin in response to stress. This approach stems from the observations made by Gomes *et al*. who showed that cohesin is evicted from the p53 target gene *CDKN1A* following 5-fluorouracil treatment, supporting that cohesin could be remodeled from its binding sites in response to stress [22]. We thus reasoned that sites where stress induces remodeling of cohesin might be good candidate loci were chromatin loops could be rearranged during the p53 stress response, *i*.*e*. chromatin loops might be disrupted when cohesin is evicted from its binding sites. Hence, we used the cohesin subunit Rad21 as a proxy for cohesin and performed a ChIP-seq of Rad21 in HCT116 p53^+/+^ cells treated with the DNA damaging agent daunorubicin. RNA-seq experiments were carried out in parallel to obtain the transcription level of genes following stress induction. The biological replicates of these experiments exhibited a high degree of correlation (Pearson’s r > 0.97, S1A and S1B Fig) and were thus pooled for the bioinformatics analysis.

**Fig 1:**
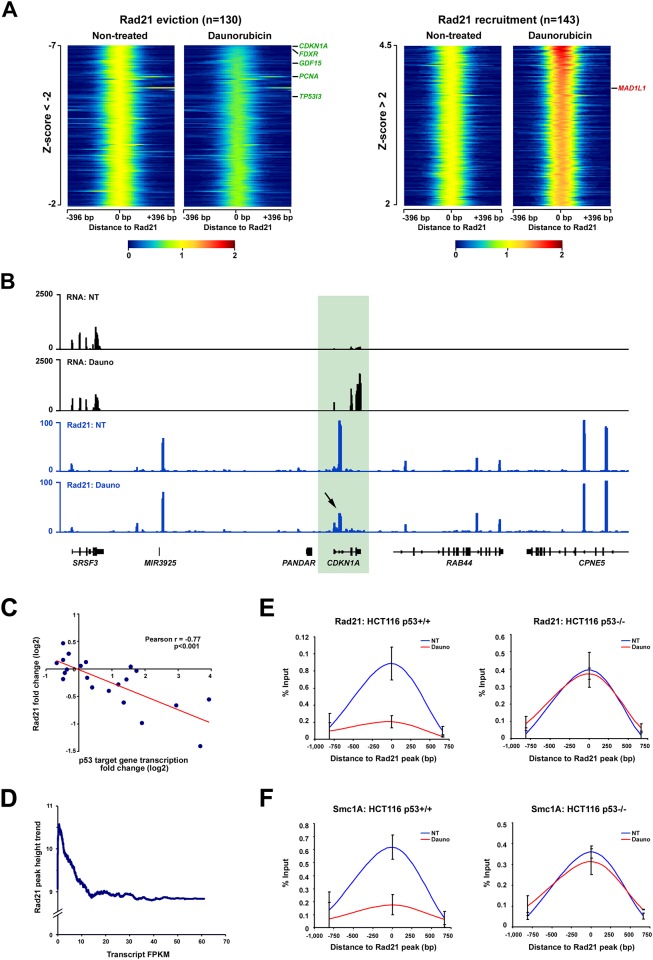
Cohesin binding is remodeled in response to stress and negatively correlates with the transcription induction of p53 target genes. (**A**) Heatmaps of Rad21 signal measured by ChIP-seq in non-treated and daunorubicin treated HCT116 p53^+/+^ cells. Rad21 binding sites with a log2 fold change Z-score inferior to -2 and superior to 2 are presented. p53 target genes harboring a Rad21 site within their gene bodies and for which p53 is an activator are shown in green, those for which p53 is a repressor are shown in red (**B**) RNA-seq and Rad21 ChIP-seq tracks obtained in non-treated (NT) and daunorubicin-treated (Dauno) HCT116 p53^+/+^ cells. The signal obtained at the *CDKN1A* locus is presented. (**C**) Correlation of Rad21 binding signal fold change with gene transcription fold change for Rad21 binding sites located within p53 target genes. (**D**) Correlation of Rad21 peak height and gene transcription FPKM for all Rad21 binding sites located within genes. Data obtained in non-treated HCT116 p53^+/+^ cells. (**E**) ChIP of Rad21 in HCT116 p53^+/+^ and p53^-/-^ cells at the intragenic *CDKN1A* cohesin binding site. (**F**) ChIP of Smc1A in HCT116 p53^+/+^ and p53^-/-^ cells at the intragenic *CDKN1A* cohesin binding site.

To analyze the ChIP-seq data, we calculated the log2 fold-change of Rad21 signals following daunorubicin treatment for each Rad21 binding site and determined the corresponding Z-score values. We next displayed the signal of Rad21 binding sites according to their log2 fold change Z-score on heatmaps (Fig 1A); the lower the Z-score, the higher the eviction is and vice versa. We observed that the stress response induces Rad21 binding changes with Z-score <-4 or >4 at several sites, indicating that these changes in binding activity are highly specific and restricted to selected loci (Fig 1A). Rad21 binding was similarly affected at sites located outside or inside genes, and also inside introns or exons (S1C Fig, and S8 Table). Amongst the Rad21 binding sites located within genes, several resided within p53 target genes (Fig 1A). Strikingly, p53 target genes known to be induced by p53 in response to stress (green) exhibited a decrease in Rad21 binding from their gene body, with the cohesin site located within the *CDKN1A* gene exhibiting the highest decrease (Fig 1A and 1B and S1D Fig) [1]. The opposite was observed for target genes repressed when the p53 pathway is activated (red) (Fig 1A) [1, 23, 24]. We thus reasoned that Rad21’s eviction from p53 target gene bodies in response to stress might be coupled to transcription induction. To test this idea, we plotted fold changes in Rad21 binding obtained at sites located within p53 target genes against the fold changes in transcription of their respective genes (Fig 1C). These results revealed that there is a correlation between transcription induction levels of p53 target genes and Rad21 eviction from their gene bodies (Fig 1C, Pearson’s r = -0.77, p<0.001). This eviction was specific, as the binding of Rad21 at sites surrounding p53 target genes remained virtually unchanged in the absence of transcription (Fig 1B, S1D and S1E Fig). Additionally, the correlation between transcription induction and Rad21 eviction was also observed when the analysis was performed on all Rad21 binding sites residing within gene bodies (S1F Fig). Next, we speculated that if Rad21 binding site occupancy negatively correlates with transcription, a corollary would be that the height of Rad21 peaks located within highly transcribed genes has to be lower than in weakly transcribed genes. This was confirmed when we plotted the height of Rad21 peaks located within genes against the Fragments Per Kilobase of exon per Million (FPKM) of their respective genes (Fig 1D and S1G Fig). We thus conclude that the binding of Rad21 within gene bodies negatively correlates with transcription.

To verify that the eviction of Rad21 in response to stress is coupled to the transcriptional activity elicited by p53 and not caused by the stress *per se*, we performed a Chromatin Immuno-Precipitation (ChIP) of Rad21 in HCT116 p53^+/+^ and HCT116 p53^-/-^ cells treated or not with daunorubicin. We measured the binding of Rad21 at the *CDKN1A (p21)* site because *CDKN1A* induction in response to daunorubicin is virtually abolished in HCT116 p53^-/-^ cells (S1H Fig). This experiment clearly demonstrates that daunorubicin induces Rad21 eviction exclusively in the HCT116 p53^+/+^ cells, thus confirming that p53 transcriptional activity is required to evict Rad21 from the *CDKN1A* body (Fig 1E). Similar results were obtained when we monitored the Smc1A cohesin subunit, thus supporting the fact that the data obtained with Rad21 reflect the fate of the entire cohesin complex (Fig 1F). Finally, S2 Fig shows that these observations can also be generalized to other stress-induced p53 target genes possessing a cohesin binding site within their gene bodies, further supporting that the entire cohesin complex is evicted from the body of p53 target genes following gene induction.

Altogether, these data demonstrate that cohesin binding is altered in response to stress at defined loci. They also show that cohesin binding within gene bodies tends to negatively correlates with gene transcription. This is exemplified at p53 target genes harboring cohesin binding sites within their gene bodies, where cohesin eviction is highly correlated with transcription induction. Because cohesin plays a role in the formation of DNA interactions, we reasoned that p53 target genes that exhibit cohesin remodeling from their gene bodies in response to stress could be potential sites for chromatin loop disruption and changes in local spatial genome architecture.

### The spatial architecture of p53 target genes is reorganized in response to stress and coincides with cohesin eviction form their gene bodies

To investigate whether p53 target genes undergo spatial rearrangement in response to stress, we analyzed the architecture of p53 target genes that exhibit cohesin eviction from their gene bodies following daunorubicin treatment. We first started by studying the locus architecture of *CDKN1A*, which we found to be the p53 target gene with an intronic cohesin site showing the highest decrease in Rad21 binding in response to stress (Fig 1A and S8 Table).

As a first step, we queried potential functional elements of the *CDKN1A* gene that could be involved in DNA interactions. Two obvious elements to consider were the promoter and the newly identified intragenic cohesin site. However, we also took into account the fact that cohesin often colocalizes with CTCF to form insulator sites that typically separate functional DNA elements [15, 25]. Thus, the cohesin site located within *CDKN1A* could possibly be an insulator that separates the *CDKN1A* promoter from a downstream functional element, as suggested by the presence of CTCF at this locus in several cell lines (S3A Fig). To explore this possibility, we performed a ChIP to probe for the presence of CTCF in HCT116 cells (S3B Fig). This experiment confirmed that CTCF is present at the cohesin site, which indicates that this site could indeed be an insulator. Subsequently, to test if this cohesin-CTCF site actually acts as an insulator for histone marks, we performed ChIP to measure the level of H3K4me1 and H3K4me3. Typically, a ratio of H3K4me3>H3K4me1 is found at promoter regions, while a ratio of H3K4me3<H3K4me1 is characteristic of enhancers or alternative internal promoters [26–28]. S3C Fig shows that the cohesin-CTCF site behaves as a sharp insulator for those histone marks and divides the *CDKN1A* gene into two regions. Upstream from the cohesin-CTCF site, an H3K4me3/H3K4me1 ratio typical of promoters was observed in the vicinity of the *CDKN1A* transcription start site (TSS) [26]. Conversely, the region downstream from the cohesin-CTCF site was characterized by an H3K4me3/H3K4me1 ratio typical of enhancers or alternative internal promoters [26–28]. Next, we investigated if a functional element could be located within this region by probing for the presence of a nucleosome depleted region (NDR), a hallmark of active chromatin domains, using Formaldehyde-Assisted Isolation of Regulatory Elements (FAIRE) [29]. FAIRE revealed a NDR at +2,000 bp from the cohesin-CTCF site that correlates with a DNaseI sensitive peak when aligned with the ENCODE DNaseI track (S3C and S3D Fig) [30]. We thus considered three functional elements for investigating the spatial architecture of the *CDKN1A* locus in response to stress: 1) the intragenic cohesin site, 2) the *CDKN1A* promoter, and 3) the NDR located downstream from the cohesin site (S4A Fig).

**Fig 2:**
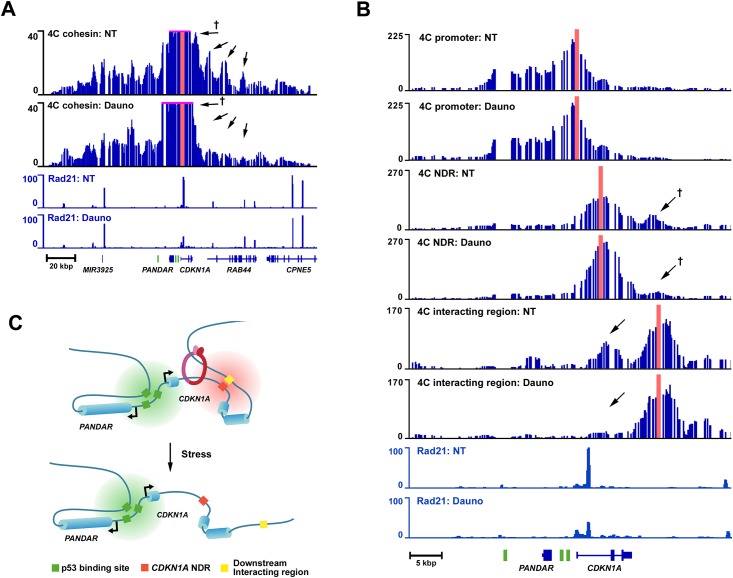
The spatial organization of the *CDKN1A* locus is remodeled in response to stress. (**A**) 4C high-resolution interaction map of *CDKN1A*’s intragenic cohesin site. HCT116 p53^+/+^ cells were treated (Dauno) or not (NT) with daunorubicin. The 4C cohesin site viewpoint is highlighted in red; Rad21 ChIP-seq tracks are also shown. P53 binding sites are indicated in green in the gene track. (**B**) 4C high-resolution interaction map of the *CDKN1A* locus. HCT116 p53^+/+^ cells were treated (Dauno) or not (NT) with daunorubicin. The 4C tracks for the promoter, nucleosome-depleted region (NDR), and interacting region are presented with their respective viewpoints in red. Rad21 ChIP-seq tracks are also shown. P53 binding sites are shown in green in the gene track. (**C**) Model of the dynamic spatial organization of the *CDKN1A* locus in response to stress. (**†**) Indicates the same 4C peak between between (**A**) and (**B**).

We then studied the spatial organization of the *CDKN1A* locus in response to stress using high-resolution 4C (S4A Fig) [31, 32]. This technique identifies short-range DNA interactions at high resolution. We first measured the DNA interactions occurring at the intragenic cohesin site in non-treated and daunorubicin-treated HCT116 p53+/+ cells (Fig 2A). The high-resolution 4C-seq revealed that *CDKN1A*’s architecture is remodeled in response to stress and is divided into two interacting regions. Upstream from the cohesin sites, the interactions occurring up to the *SRSF3* gene remain globally unchanged following stress (Fig 2A). In stark contrast, the interactions occurring downstream from the cohesin site are highly dynamic and are disrupted in response to stress (Fig 2A, see arrows). High-resolution 4C-seq performed at the *CDKN1A* promoter and the *CDKN1A* NDR lent further support to this organization of the locus (Fig 2B). The *CDKN1A* promoter, which is upstream from the cohesin site, forms a broad interacting region that remains stable under stress and encompasses three p53 binding sites and the co-regulated non-coding RNA “Promoter Of *CDKN1A* Antisense DNA Damage Activated RNA” (*PANDAR*) (Fig 2B, see promoter tracks) [33–35]. On the other hand, the interactions occurring at the *CDKN1A* NDR, which is located downstream to the cohesin site, are highly dynamic in response to stress. In the absence of stress, the NDR interacts with a region downstream from *CDKN1A* (Fig 2B, see NDR NT track). Strikingly, following an 8h daunorubicin treatment, the interaction between the NDR and the region downstream from *CDKN1A* is virtually abolished (Fig 2B, see NDR Dauno track). To confirm the existence of this stress-dependent chromatin loop, we performed both a biological and technical replicate of this experiment, as well as a 4C experiment using the interacting region as a viewpoint (S4B Fig and Fig 2B Interacting region tracks). These experiments were in agreement with our initial observations. Additionally, to rule out a bias related to the type of first cutter enzyme used, we performed a 4C experiment with Csp6I as first cutter instead of DpnII (S4C Fig). Importantly, we obtained similar results to those obtained with DpnII, which confirms the interaction and its dynamic nature. Lastly, we tested whether the disruption of this stress-dependent chromatin loop is coupled to the transcriptional activation of *CDKN1A*. We carried out a 4C experiment in HCT116 p53^-/-^ cells, for which daunorubicin treatment does not induce p53-dependent *CDKN1A* transcription (S1H and S4D Figs). The experiment revealed that, in the absence of *CDKN1A* transcription, the interaction between the NDR and the region downstream of *CDKN1A* is not disrupted and remains stable (S4D Fig). Based on these data we conclude that the *CDKN1A* locus has a pre-established spatial architecture, which is partially disrupted concomitantly to cohesin eviction from the *CDKN1A* gene body (Fig 2C). Because the cohesin site also interacts in a stress dependent manner with the downstream interacting region (Fig 2 arrows marked with crosses and S5 Fig tracks 4C cohesin) and because the cohesin site is part of the interaction between the NDR and the downstream interacting region (S5 Fig tracks 4C interacting region), we propose that cohesin might be involved in the formation and maintenance of the dynamic loop observed at *CDKN1A* (Fig 2C).

**Fig 3:**
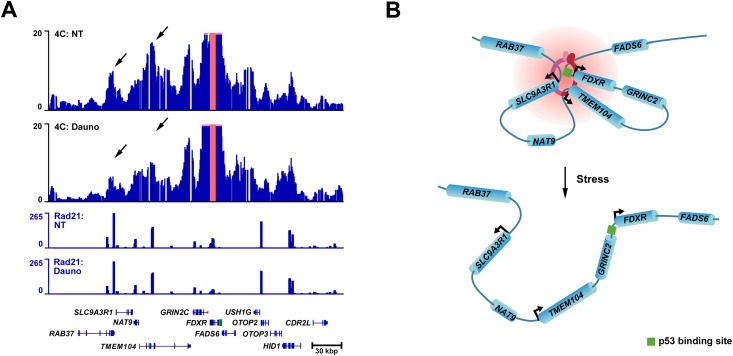
The spatial organization of the *FDXR* locus is remodeled in response to stress. (**A**) 4C high-resolution interaction map of the *FDXR* intragenic cohesin site. HCT116 p53^+/+^ cells were treated (Dauno) or not (NT) with daunorubicin. The 4C cohesin site viewpoint is highlighted in red; Rad21 ChIP-seq tracks are also shown. p53 binding sites are shown in green in the gene track. (**B**) Model of the dynamic spatial organization of the *FDXR* locus in response to stress.

We next investigated whether the disruption of chromatin loops observed at the *CDKN1A* locus in response to stress is a feature shared by other p53 target genes harboring cohesin sites within their gene bodies. To do so, we studied the spatial organization of *FDXR*, a gene that encodes a protein that helps sensitize cells to ROS-mediated apoptosis [36]. *FDXR* contains a cohesin binding site within its 6^th^ intron where cohesin is strongly evicted in response to stress (Fig 1A and S1D Fig) [36, 37]. We thus performed a high-resolution 4C experiment and measured the interaction occurring at the *FDXR* cohesin site before and after stress treatment (Fig 3A). We observed that in the absence of stress, *FDXR* interacts with the promoters of the *SLC9A3R1* and *TMEM104* genes (Fig 3A). Following stress treatment, the interaction of *FDXR* with these two gene promoters is severely compromised. We conclude that, similarly to what was observed at *CDKN1A*, the *FDXR* locus architecture is remodeled in response to stress and chromatin loops are disrupted concomitantly to cohesin eviction from the body of *FDXR* (Fig 3B).

Altogether, these data demonstrate that the spatial architecture of p53 target genes is remodeled in response to stress concomitantly to cohesin eviction from their gene bodies (Figs 2C and 3B). This clearly establishes that chromatin loops are remodeled during the p53 stress response program. These results prompted us to test whether the remodeling of stress-dependent chromatin loops is important for the regulation of p53 target genes since it could control interactions between functional DNA elements.

### The *CDKN1A* NDR stress-dependent chromatin loop controls the interaction between a *CDKN1A* alternative internal promoter and a downstream repressor element

**Fig 4:**
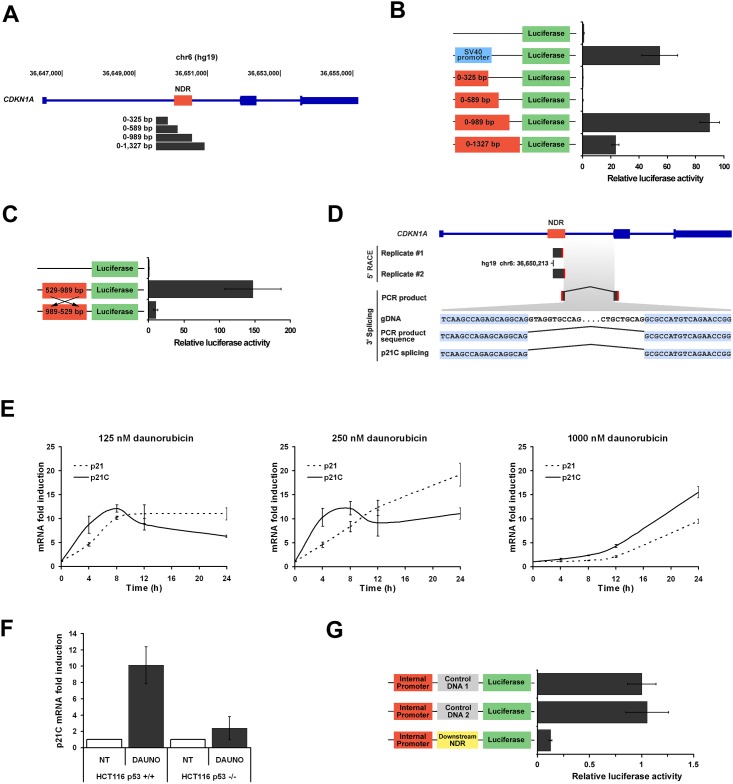
The *CDKN1A* stress-dependent chromatin loop controls the interaction between a *CDKN1A* alternative internal promoter and a downstream repressor element. (**A**) *CDKN1A* gene with the DNA fragments spanning the *CDKN1A* nucleosome-depleted region (NDR) cloned for the luciferase assay. Base counting starts at position chr6:36,649,572 strand (+) hg19. (**B**) Luciferase reporter assay to measure the promoter activity of the *CDKN1A* NDR. Signal was normalized to the empty vector. (**C**) Luciferase reporter assay to test the directionality of the putative promoter. Signal was normalized to the empty vector. (**D**) Experiments to locate the TSS and the 3’ splicing junction of the mRNA transcribed from the internal promoter. The 5’ RACE products from two biological replicates obtained using two different sets of primers were sequenced and aligned to the *CDKN1A* gene (S9 Table). The 3’ splicing was determined by performing a PCR on cDNA using a forward primer overlapping the 5’ RACE product and a reverse primer located on exon 2 of p21. The sequencing results of the PCR are shown along with the p21C splicing sequence. Experiments were performed in HCT116 p53^+/+^ cells treated with daunorubicin. Red rectangles correspond to primers. (**E**) p21 and p21C mRNA levels assayed by RT-qPCR in HCT116 p53^+/+^ cells exposed to 125, 250, or 1000 nM daunorubicin and collected over a 24 h time course. (**F**) p21C mRNA level assayed by RT-qPCR in HCT116 p53^+/+^ and p53^-/-^ cells exposed to 250 nM daunorubicin for 8 h. (**G**) Luciferase reporter assay to test the repressor activity of the interacting region on the *CDKN1A* internal promoter. The luciferase signal is normalized to Control DNA1.

To determine whether the stress-dependent chromatin loop observed at the *CDKN1A* NDR is important for the regulation of this locus, we started by investigating the nature of the *CDKN1A* NDR (S3C and S3D Fig). Based on recent evidence that intragenic regions harboring enhancer histone marks can also act as alternative promoters, we tested whether the *CDKN1A* NDR presented any promoter activity [28]. DNA fragments spanning the *CDKN1A* NDR region were tested for potential promoter activity in a luciferase reporter assay (Fig 4A). Strong luciferase activity was obtained with the DNA sequence comprised between 589 bp and 989 bp, supporting the fact that a putative promoter is located within this region (Fig 4B and S3C Fig). In a reverse orientation, the 529–989 bp fragment no longer exhibited any promoter activity (Fig 4C) [38]. Next, to test if this promoter is regulated in response to stress, we compared its activity before and after daunorubicin treatment; no changes were observed (S6A Fig). Similarly, the promoter activity obtained in HCT116 p53^+/+^ cells was virtually identical to the one obtained in HCT116 p53^-/-^, indicating that the promoter is not p53-dependent (S6B Fig). Based on these data, we propose that the *CDKN1A* NDR is an internal unidirectional promoter whose activity does not seem to be regulated by stress or p53, at least not in the context of a reporter gene assay.

We next wanted to test whether this putative promoter is actually a functional internal promoter in HTC116 cells. First, we determined if a TSS is located within the *CDKN1A* NDR by performing a 5’ Rapid Amplification of cDNA Ends (RACE) in HCT116 cells treated with daunorubicin. Fig 4D shows that a TSS is present at position chr6:36,650,213 strand (+) hg19, which overlaps with the *CDKN1A* NDR and the 589–989 bp DNA fragment. We next investigated the nature of the transcript starting from this internal promoter. Nozell and Chen previously reported that p21C, a p21 variant, is transcribed from intron 1 of *CDKN1A*. p21C differs from p21 mRNA by its first non-coding exon that splices with the exon 2 of p21 [39]. However, it shares the same coding region as p21 and, consequently, also encodes the same p21 protein. Because exon 1 of p21C overlaps with the *CDKN1A* NDR, we speculated that the internal promoter might be responsible for the expression of p21C. Therefore, we determined the 3’ splice junction between the 5’ RACE product and the exon 2 of p21. A PCR using a forward primer overlapping the 5’ RACE product and a reverse primer located within exon 2 of p21 confirmed that the 5’ RACE product is spliced with exon 2 of p21 (Figs 4D and S6C). Subsequent sequencing of this PCR product established that the splicing junction is identical to p21C’s, supporting the notion that the internal promoter is responsible for the expression of the p21C variant transcript (Fig 4D). Next, we used RT-qPCR to compare p21C and p21’s expression in response to different doses of daunorubicin (Fig 4E). Strikingly, mRNA levels of p21C increased very rapidly compared to those of p21, which indicates that p21 and p21C mRNA levels are regulated differently in response to stress. Finally, we assessed by RT-qPCR whether p21C is expressed in HCT116 p53^-/-^ cells (Fig 4F). The expression of p21C was severely impaired in HCT116 p53^-/-^ cells compared to HCT116 p53^+/+^ cells, indicating that p21C expression is directly dependent on p53 or coupled to *CDKN1A* activation. Based on these observations, we propose that the *CDKN1A* NDR is an internal promoter that controls the expression of p21C and acts as an alternative promoter for the regulation of *CDKN1A* in response to stress.

Finally, we sought to investigate if a regulatory element was located within the downstream interacting region, and if so, to elucidate its role in the regulation of the *p21C* promoter (Fig 2C). We first assessed whether a NDR was located within the interacting region. Alignment of the high-resolution 4C-seq data with the DNaseI tracks from the ENCODE project revealed that a DNaseI sensitive site is located near the 4C interaction peak and FAIRE experiments confirmed that this NDR is also present in HCT116 cells (S7A and S7B Fig) [30]. In order to determine the function of this putative regulatory element, we again made use of luciferase reporter assays to test its distal and proximal enhancer activity, as well as its promoter activity (S7C, S7D and S7E Fig). Regardless of the activity tested, this NDR failed to increase the level of luciferase, which eliminates any possible enhancer or promoter function. We next considered the possibility that the downstream NDR might be a repressor element. We first tested if the downstream NDR could repress enhancer activities and inserted it between the SV40 enhancer and the SV40 promoter (S7F Fig). No repression of transcription was noted. We next tested if the downstream NDR could interfere with the transcription machinery of the internal promoter. We inserted it between the internal promoter and the luciferase reporter (Fig 4G). Remarkably, in this context, the NDR efficiently repressed the internal promoter activity up to eight-fold when compared to two control DNA regions. We thus conclude that the interacting region contains a regulatory element capable of inhibiting the activity of the *CDKN1A* alternative internal promoter.

Taken together, our results demonstrate that the stress-dependent chromatin loop observed at *CDKN1A* is functional and controls the interaction of a putative repressor element with an alternative internal promoter responsible for the expression of the p21C transcript.

## Discussion

The establishment of p53’s stress response program is a crucial mechanism for preventing carcinogenesis. This process requires the rapid and coordinated regulation of hundreds of p53 target genes. Here we unveil that the spatial genome architecture is locally remodeled at specific loci during the stress response, which is accompanied by changes in gene regulation. This is illustrated at p53 target genes where eviction of cohesin from gene bodies in response to stress coincides with the disruption of functional chromatin loops and spatial reorganization.

Our data reveal that specific loci exhibit profound alteration in Rad21 binding following stress treatment. This happens equally at sites located outside genes or within introns and exons. However, we show that for Rad21 sites located within genes there is a correlation between the binding of Rad21 and the transcriptional status of the gene. For genes induced in response to stress, Rad21 binding decreases as gene expression increases, which is noticeable at p53 target genes (Fig 1C). In the absence of p53, stress *per se* is not sufficient to induce cohesin eviction, suggesting that the activity of p53 is required to evict cohesin (Fig 1E and 1F). At least two mechanisms might explain how p53 activity could result in cohesin eviction from target gene bodies. A first possibility is that p53 directly recruits a cohesin-releasing protein to cohesin binding sites (*e*.*g*. Wapl) [14]. However, as p53 ChIP-seq data clearly shows that p53 and cohesin binding sites do not overlap, this mechanism seems unlikely (S8 Fig) [40]. Rather, we favor a mechanism where p53-induced transcription causes local eviction of cohesin, similar to the removal of histone proteins from gene bodies upon passage of RNA polymerase II [41, 42]. This model is supported by two observations. First, the eviction of cohesin from p53 target gene bodies positively correlates with the level of transcriptional induction (Fig 1C). Second, even in non-stressed cells, Rad21 peak heights decrease as genic transcription levels increase, regardless of whether genes are regulated by p53 or not (Fig 1D). Both pieces of evidence suggest that the presence of cohesin within genes correlates negatively with transcription. Therefore, we propose that cohesin eviction from p53 target gene bodies could be the consequence of the transcriptional induction triggered by p53, and the subsequent elongation activity of the RNA polymerase II machinery.

The structural data obtained at the p53 target genes *CDKN1A* and *FDXR* using high-resolution 4C reveals a correlation between cohesin eviction and the disruption of chromatin loops. We show that in non-stressed cells the *CDKN1A* locus is shaped by pre-established chromatin loops: a large interacting hub connects the *CDKN1A* promoter, *PANDAR*, and a cluster of three p53 binding sites, while the *CDKN1A* alternative internal promoter for p21C interacts solely with a downstream repressor element (Fig 2B and 2C). Following cohesin eviction in response to stress, the large interacting hub occurring at the promoter remains stable, while the chromatin loop connecting the internal promoter and the downstream repressor element is disrupted (Fig 2B and 2C). Similar chromatin loop dynamics were observed at the *FDXR* locus in response to stress. Upon eviction of cohesin from intron 6 of *FDXR* following gene induction, the interactions occurring with the *SLC9A3R1* and *TMEM104* genes are disrupted (Fig 3A and 3B). These results clearly establish that chromatin loops are remodeled during the p53 stress response program and that eviction of cohesin coincides with the disruption of functional chromatin loops.

The high-resolution 4C-Seq data also reveal insights on the link between the spatial organization of p53 target gene loci and their role in the stress response. In the case of the *CDKN1A* locus, the fact that the *CDKN1A* promoter, *PANDAR*, and the p53 binding sites cluster form a large interacting hub in non-stressed cells could be advantageous for regulating this locus in response to stress. Indeed, this pre-established hub allows stress activated p53 to bind to response elements already engaged in interactions with promoters, resulting in a faster upregulation of genes, in this case, *CDKN1A* and *PANDAR* [43]. Since *CDKN1A* and *PANDAR* are both involved in the anti-apoptotic and cell-cycle arrest response axis, this hub provides a unique way to “share” the activity of several p53 binding sites in order to co-regulate functionally complementary genes. Similarly, at the *FDXR* locus, *FDXR*, *TMEM104*, and *SLC9A3R1* also form an interacting hub, suggesting that an interdependent regulation of these genes could also occur (Fig 3A). In fact, functionally complementary genes are interacting together as *TMEM104* and *SLC9A3R1* are both involved in the biology of the cellular membrane, and *FDXR* and *SLC9A3R1* are involved in the induction of apoptosis [36, 44–48]. We propose that this hub might be important for the control of apoptosis, although the contribution of *TMEM104* to this process remains to be determined as its function has not yet been deciphered. As observed with *CDKN1A*, the analysis of the chromatin loops occurring at the *FDXR* locus reveals a unique architecture that provides a way to control a cluster of functionally complementary genes. Our data thus indicates that p53 target genes could be organized into genomic units that include multiple genes involved in the same biological pathways for efficient gene regulation.

Finally, on a functional level, we show that the role of these stress-dependent chromatin loops might be important for the fine-tuning of the p53 stress response transcriptional program. At the *CDKN1A* locus, we reveal that the stress-dependent chromatin loop controls the interaction between the *p21C* promoter and a repressor element. In non-stressed cells, the internal promoter is repressed through the action of the downstream repressor element, while in response to stress the interaction is disrupted following *CDKN1A* activation and allows transcription of *p21C* from the internal promoter (Figs 2B and 4D). Since this variant shares the same open reading frame as *p21*, and thus encodes the same protein, the biological function of p21C is the same as p21 [39]. However, we show that its expression profile differs from that of *p21*; the accumulation of *p21C* mRNA in response to stress is much faster than for *p21* (Fig 4E). Because p21C mRNA has a different 5’-UTR than p21 mRNA, this could affect positively its stability and explains its accumulation level. We propose that the expression of p21C variant might provide an alternative way to quickly produce the p21 protein independently from the main promoter and contribute to the rapid establishment and fine tuning of the p53 stress response.

**Fig 5:**
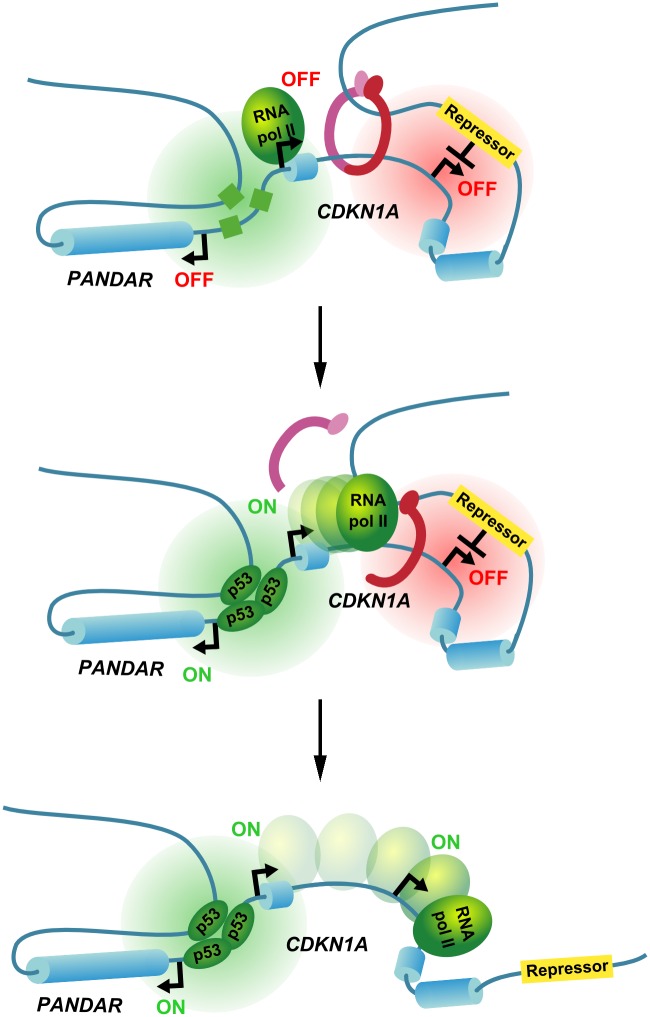
Model of the *CDKN1A* chromatin loop molecular switch. Following activation of *CDKN1A* by p53 in response to stress, the passage of the transcriptional machinery evicts the cohesin and disrupts the chromatin loop. This releases the internal promoter from the influence of the repressor element and results in the induction of p21C.

To conclude, we propose a model where the eviction of cohesin from gene bodies following gene activation could operate as a molecular switch to control interactions between regulatory elements and target promoters. As exemplified at *CDKN1A*, this mechanism would provide an efficient way to co-regulate genes based on the structural changes in the local genome architecture induced by the transcriptional activation of a “master” gene (Fig 5).

## Supporting Information

S1 Fig Rad21 binding is remodeled in response to stress and negatively correlates with transcription induction of p53 target genes. (**A**) Correlation between Rad21 ChIP-seq biological replicates. (**B**) Correlation between RNA-seq biological replicates. (**C**) Distribution of Rad21 ChIP-seq peaks with a Z-score <-2 and >2 within the genome. (**D**) RNA-seq and Rad21 ChIP-seq tracks obtained in HCT116 p53+/+ non-treated (NT) or treated with daunorubicin (Dauno) for the *FDXR* locus. (**E**) ChIP-seq signal of Rad21 binding sites surrounding the *CDKN1A* and *FDXR* genes. When Rad21 sites are located within genes, the transcription level fold induction of the gene following daunorubicin is indicated. (**F**) Correlation of Rad21 binding signal fold change with gene transcription fold change for Rad21 binding sites located within genes. (**G**) Correlation of Rad21 peak height and gene transcription FPKM for Rad21 binding sites located within genes. Data obtained in daunorubicin treated HCT116 cells. (**H**) p21 mRNA level assayed by RT-qPCR in HCT116 p53 +/+ and p53 -/- cells treated or not with daunorubicin.
(DOC)

S2 Fig Rad21 and Smc1A are evicted from the body of the stress induced p53 target genes *FDXR*, *TP53i3* and *GDF15*. (**A**) mRNA induction in response to stress measured by RNA-seq for the p53 target genes *FDXR*, *TP53i3*, and *GDF15* (**B**) ChIP of Rad21 and Smc1A at cohesin binding sites located within gene bodies of *FDXR*, *TP53i3*, and *GDF15* in HCT116 p53+/+. (**C**) ChIP of Rad21 and Smc1A at cohesin binding sites located within gene bodies of *FDXR*, *TP53i3*, and *GDF15* in HCT116 p53-/-.
(DOC)

S3 Fig A nucleosome depleted region is located in the intron 1 of *CDKN1A* downstream to the cohesin site. (**A**) The CTCF-Cohesin site present within the first intro of *CDKN1A* is conserved between different cell types. CTCF and Rad21 ChIP-seq signal at the *CDKN1A* gene is shown for different cell types. Representation adapted from the UCSC genome browser using tracks from the ENCODE project. (**B**) ChIP of CTCF and Rad21 performed in HCT116 p53+/+ cells. The signal obtained at the *CDKN1A* cohesin site is shown. (**C**) FAIRE experiment and ChIP experiment of H3K4me1, H3K4me3, and H3 carried out in non-treated HCT116 cells. H3K4me1 and H3K4me3 ChIP results were normalized to H3 to account for nucleosome density. (**D**) *CDKN1A* gene with the nucleosome depleted region shown in red. The DNaseI 125 cell types track (ENCODE data from the University of Washington ENCODE group on behalf of the ENCODE Analysis Working Group), and the ChIP-seq track for Rad21 obtained in HCT116 p53+/+ non-treated are also shown. Adapted from the UCSC genome browser.
(DOC)

S4 Fig The 3D spatial organization of the *CDKN1A* locus is remodeled in response to stress. (**A**) Representation of the *CDKN1A* gene with the promoter, the cohesin, and the NDR 4C viewpoints. 4C DpnII restriction sites are shown in red. The ENCODE DNaseI tracks (data from the University of Washington ENCODE group on behalf of the ENCODE Analysis Working Group) adapted from the UCSC genome browser is also shown. (**B**) Biological and technical replicate of the high-resolution 4C experiment carried out in HCT116 cells treated or not with daunorubicin using DpnII as first cutter. The NDR viewpoint track is shown. (**C**) High-resolution 4C experiment performed using Csp6I as first cutter (instead of DpnII) in HCT116 cells treated or not daunorubicin. The NDR viewpoint track is shown. (**D**) High-resolution 4C experiment carried out in HCT116 p53-/- cells treated or not with daunorubicin using DpnII as first cutter. The NDR viewpoint track is shown.
(DOC)

S5 Fig The *CDKN1A* cohesin site is part of the interaction between the NDR and the downstream interacting region. 4C high-resolution interaction map of the *CDKN1A* locus. HCT116 p53^+/+^ cells were treated (Dauno) or not (NT) with daunorubicin. The 4C tracks for the cohesin site and the interacting region are presented with their respective viewpoints in red. Rad21 ChIP-seq tracks are also shown. P53 binding sites are shown in green in the gene track.
(DOC)

S6 Fig The *CDKN1A* NDR is an internal alternative promoter. (**A**) Luciferase reporter assay to test if the internal promoter activity is affected by stress. (**B**) Luciferase reporter assay to test if the activity of the internal promoter is p53-dependent. (**C**) PCR products obtained with primers amplifying the 5’RACE / p21 exon 2 junction using gDNA as template or cDNA from daunorubicin treated HCT116 cell.
(DOC)

S7 Fig The *CDKN1A* downstream interacting region is a repressor element. (**A**) 4C track of the NDR viewpoint obtained in non-treated HCT116 cells aligned to the ENCODE DNaseI track (data from the University of Washington ENCODE group on behalf of the ENCODE Analysis Working Group). Adapted from the UCSC genome browser. (**B**) FAIRE experiment carried out in non-treated HCT116 cells. Primer sets used were tailing the DNaseI peak located at chr6:36,660,086–36,660,295 strand (+) hg19. (**C**) Luciferase reporter assay to test the distal enhancer activity of the interacting region regulatory element. The signal is normalized to the Internal promoter/Luciferase construction. (**D**) Luciferase reporter assay to test the proximal enhancer activity of the interacting region regulatory element. The signal is normalized to the Control DNA1/Internal promoter/Luciferase construction. (**E**) Luciferase reporter assay to test the promoter activity of the interacting region regulatory element. The signal is normalized to the Control DNA1/Luciferase construction. (**F**) Luciferase reporter assay to test if the interacting region regulatory element represses SV40 enhancer activity. The signal is normalized to the SV40 promoter/Luciferase construction.
(DOC)

S8 Fig p53 does not colocalize with Rad21. Venn diagram of the intersection of p53 binding sites and Rad21 binding sites in HCT116 p53+/+.
(DOC)

S1 File Supplementary biofinformatic procedures. (DOC)

S1 Table ChIP antibodies. (DOC)

S2 Table ChIP qPCR primers. (DOC)

S3 Table RT-qPCR primers. (DOC)

S4 Table FAIRE qPCR primers. (DOC)

S5 Table 4C primers. (DOC)

S6 Table Luciferase reporter assay genomic sequences. (DOC)

S7 Table 5’RACE primers. (DOC)

S8 Table Coordinates of cohesin binding sites with a Z-score <-2 or >2. (DOC)

S9 Table 5’ RACE sequencing results. (DOC)
