# Supplementary material for: High-Resolution 4C Reveals Rapid p53-Dependent Chromatin Reorganization of the CDKN1A Locus in Response to Stress
Source: PLoS One. 2016 Oct 14;11(10):e0163885. doi: 10.1371/journal.pone.0163885 (PMC5065170; doi:10.1371/journal.pone.0163885)
Supplement: S1 Fig — (A) Correlation between Rad21 ChIP-seq biological replicates. (B) Correlation between RNA-seq biological replicates. (C) Distribution of Rad21 ChIP-seq peaks with a Z-score <-2 and >2 within the genome. (D) RNA-seq and Rad21 ChIP-seq tracks obtained in HCT116 p53+/+ non-treated (NT) or treated with daunorubicin (Dauno) for the FDXR locus. (E) ChIP-seq signal of Rad21 binding sites surrounding the CDKN1A and FDXR genes. When Rad21 sites are located within genes, the transcription level fold induction of the gene following daunorubicin is indicated. (F) Correlation of Rad21 binding signal fold change with gene transcription fold change for Rad21 binding sites located within genes. (G) Correlation of Rad21 peak height and gene transcription FPKM for Rad21 binding sites located within genes. Data obtained in daunorubicin treated HCT116 cells. (H) p21 mRNA level assayed by RT-qPCR in HCT116 p53 +/+ and p53 -/- cells treated or not with daunorubicin. (DOC) [file pone.0163885.s001.doc]

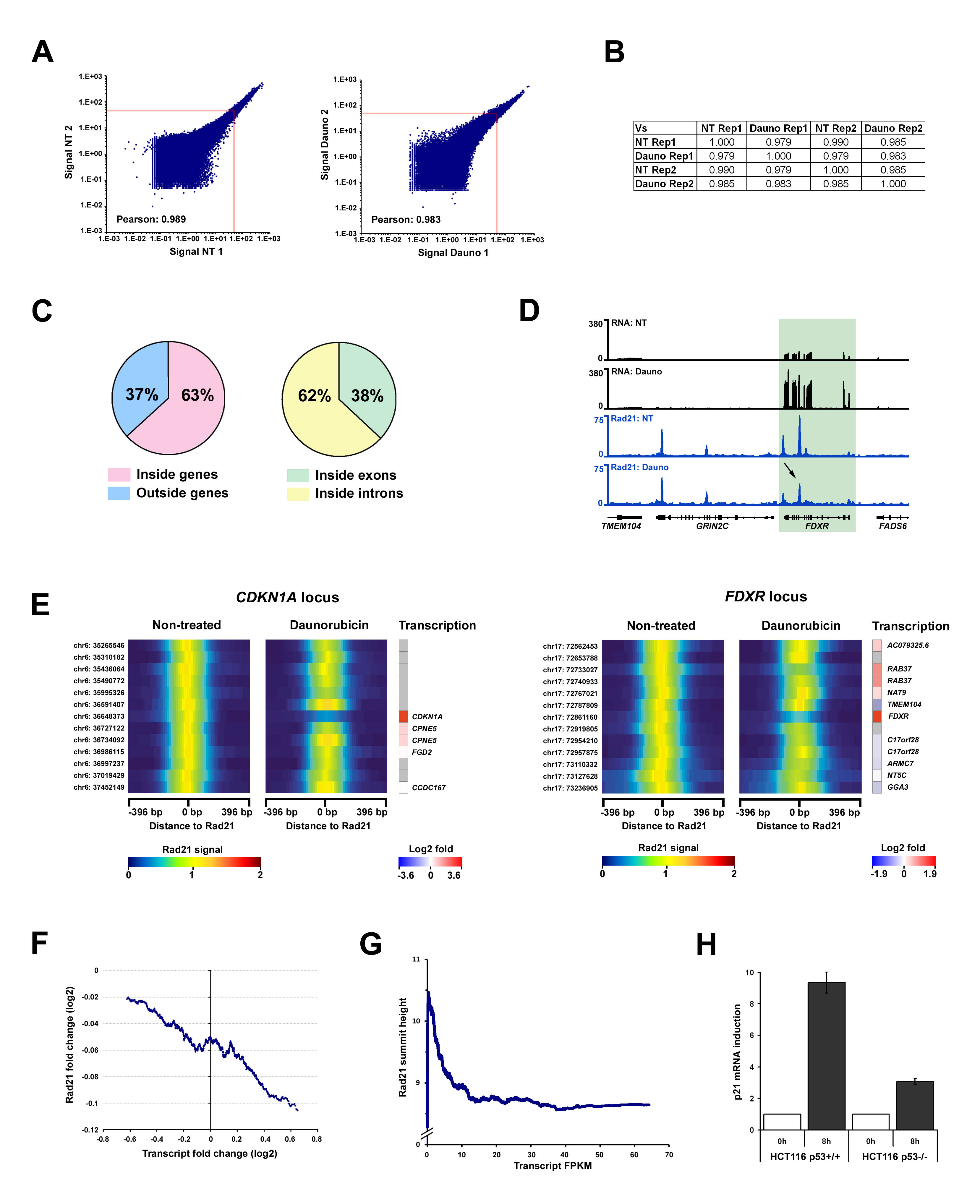


**Fig. S1**

**Figure S1. Rad21 binding is remodeled in response to stress and negatively correlates with transcription induction of p53 target genes.**

(**A**) Correlation between Rad21 ChIP-seq biological replicates. (**B**) Correlation between RNA-seq biological replicates. (**C**) Distribution of Rad21 ChIP-seq peaks with a Z-score <-2 and >2 within the genome. (**D**) RNA-seq and Rad21 ChIP-seq tracks obtained in HCT116 p53+/+ non-treated (NT) or treated with daunorubicin (Dauno) for the *FDXR* locus. (**E**) ChIP-seq signal of Rad21 binding sites surrounding the *CDKN1A* and *FDXR* genes. When Rad21 sites are located within genes, the transcription level fold induction of the gene following daunorubicin is indicated. (**F**) Correlation of Rad21 binding signal fold change with gene transcription fold change for Rad21 binding sites located within genes. (**G**) Correlation of Rad21 peak height and gene transcription FPKM for Rad21 binding sites located within genes. Data obtained in daunorubicin treated HCT116 cells. (**H**) p21 mRNA level assayed by RT-qPCR in HCT116 p53 +/+ and p53 -/- cells treated or not with daunorubicin.
