# Supplementary material for: High-Resolution 4C Reveals Rapid p53-Dependent Chromatin Reorganization of the CDKN1A Locus in Response to Stress
Source: PLoS One. 2016 Oct 14;11(10):e0163885. doi: 10.1371/journal.pone.0163885 (PMC5065170; doi:10.1371/journal.pone.0163885)
Supplement: S2 Fig — (A) mRNA induction in response to stress measured by RNA-seq for the p53 target genes FDXR, TP53i3, and GDF15 (B) ChIP of Rad21 and Smc1A at cohesin binding sites located within gene bodies of FDXR, TP53i3, and GDF15 in HCT116 p53+/+. (C) ChIP of Rad21 and Smc1A at cohesin binding sites located within gene bodies of FDXR, TP53i3, and GDF15 in HCT116 p53-/-. (DOC) [file pone.0163885.s002.doc]

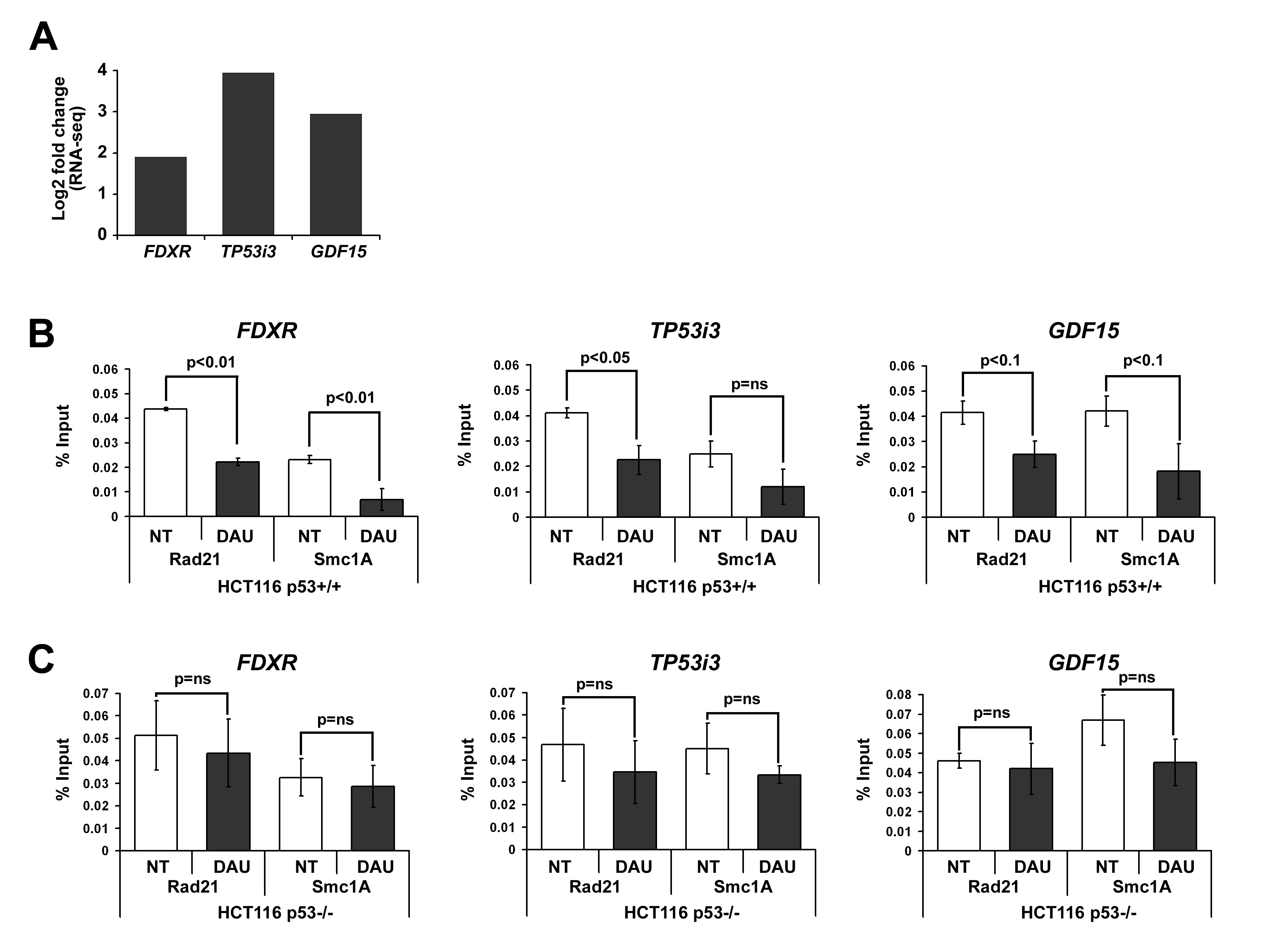


**Fig. S2**

**Figure S2. Rad21 and Smc1A are evicted from the body of the stress induced p53 target genes *FDXR*, *TP53i3* and *GDF15*.**

(**A**) mRNA induction in response to stress measured by RNA-seq for the p53 target genes *FDXR*, *TP53i3*, and *GDF15* (**B**) ChIP of Rad21 and Smc1A at cohesin binding sites located within gene bodies of *FDXR*, *TP53i3*, and *GDF15* in HCT116 p53+/+. (**C**) ChIP of Rad21 and Smc1A at cohesin binding sites located within gene bodies of *FDXR*, *TP53i3*, and *GDF15* in HCT116 p53-/-.
