# Supplementary material for: High-Resolution 4C Reveals Rapid p53-Dependent Chromatin Reorganization of the CDKN1A Locus in Response to Stress
Source: PLoS One. 2016 Oct 14;11(10):e0163885. doi: 10.1371/journal.pone.0163885 (PMC5065170; doi:10.1371/journal.pone.0163885)

**Fig. S3**

**Figure S3. A nucleosome depleted region is located in the intron 1 of *CDKN1A* downstream to the cohesin site.**

(**A**) The CTCF-Cohesin site present within the first intro of *CDKN1A* is conserved between different cell types. CTCF and Rad21 ChIP-seq signal at the *CDKN1A* gene is shown for different cell types. Representation adapted from the UCSC genome browser using tracks from the ENCODE project. (**B**) ChIP of CTCF and Rad21 performed in HCT116 p53+/+ cells. The signal obtained at the *CDKN1A* cohesin site is shown. (**C**) FAIRE experiment and ChIP experiment of H3K4me1, H3K4me3, and H3 carried out in non-treated HCT116 cells. H3K4me1 and H3K4me3 ChIP results were normalized to H3 to account for nucleosome density. (**D**) *CDKN1A* gene with the nucleosome depleted region shown in red. The DNaseI 125 cell types track (ENCODE data from the University of Washington ENCODE group on behalf of the ENCODE Analysis Working Group), and the ChIP-seq track for Rad21 obtained in HCT116 p53+/+ non-treated are also shown. Adapted from the UCSC genome browser.


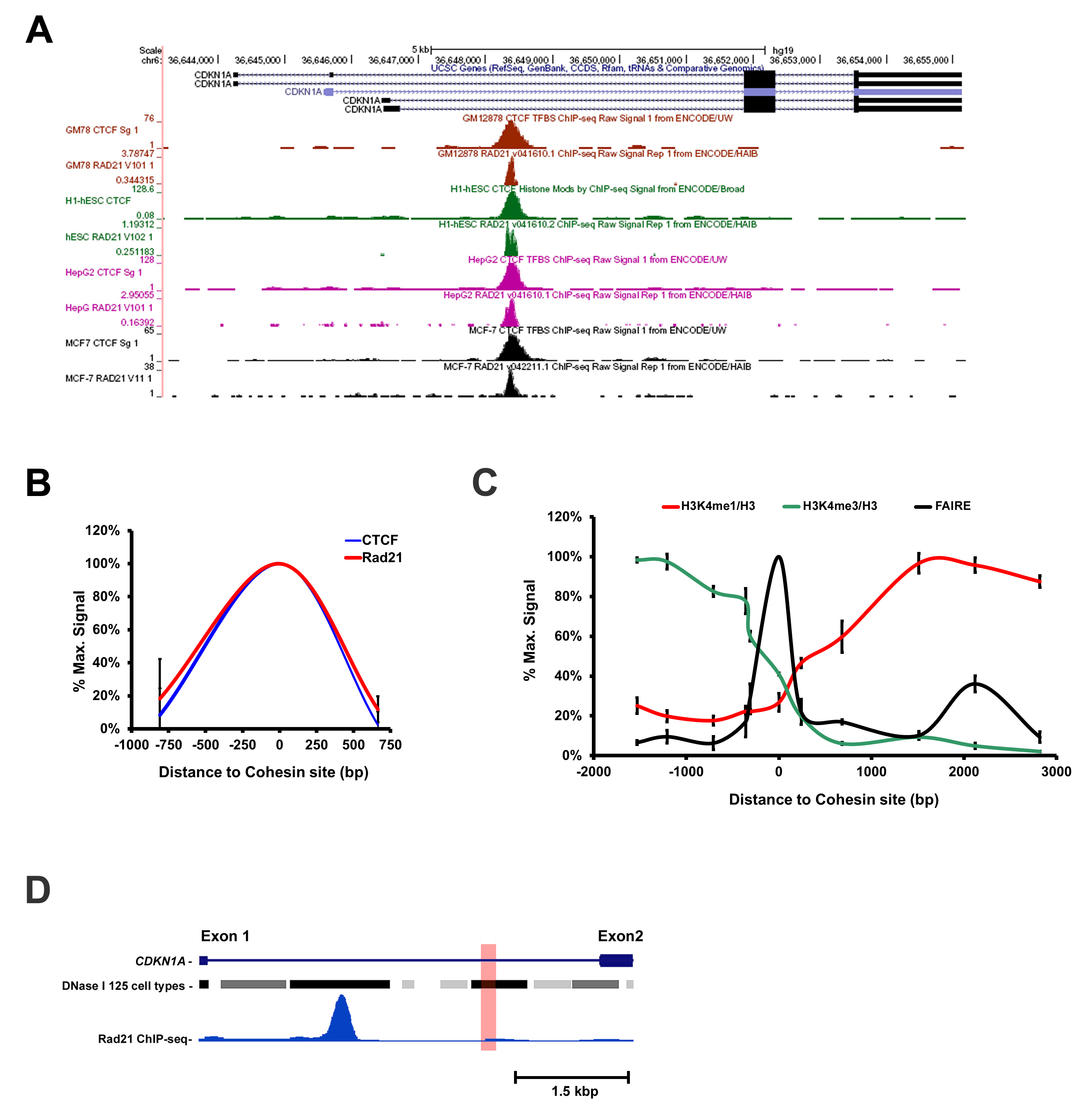

Supplement: S3 Fig — (A) The CTCF-Cohesin site present within the first intro of CDKN1A is conserved between different cell types. CTCF and Rad21 ChIP-seq signal at the CDKN1A gene is shown for different cell types. Representation adapted from the UCSC genome browser using tracks from the ENCODE project. (B) ChIP of CTCF and Rad21 performed in HCT116 p53+/+ cells. The signal obtained at the CDKN1A cohesin site is shown. (C) FAIRE experiment and ChIP experiment of H3K4me1, H3K4me3, and H3 carried out in non-treated HCT116 cells. H3K4me1 and H3K4me3 ChIP results were normalized to H3 to account for nucleosome density. (D) CDKN1A gene with the nucleosome depleted region shown in red. The DNaseI 125 cell types track (ENCODE data from the University of Washington ENCODE group on behalf of the ENCODE Analysis Working Group), and the ChIP-seq track for Rad21 obtained in HCT116 p53+/+ non-treated are also shown. Adapted from the UCSC genome browser. (DOC) [file pone.0163885.s003.doc]
