# Supplementary material for: High-Resolution 4C Reveals Rapid p53-Dependent Chromatin Reorganization of the CDKN1A Locus in Response to Stress
Source: PLoS One. 2016 Oct 14;11(10):e0163885. doi: 10.1371/journal.pone.0163885 (PMC5065170; doi:10.1371/journal.pone.0163885)
Supplement: S4 Fig — (A) Representation of the CDKN1A gene with the promoter, the cohesin, and the NDR 4C viewpoints. 4C DpnII restriction sites are shown in red. The ENCODE DNaseI tracks (data from the University of Washington ENCODE group on behalf of the ENCODE Analysis Working Group) adapted from the UCSC genome browser is also shown. (B) Biological and technical replicate of the high-resolution 4C experiment carried out in HCT116 cells treated or not with daunorubicin using DpnII as first cutter. The NDR viewpoint track is shown. (C) High-resolution 4C experiment performed using Csp6I as first cutter (instead of DpnII) in HCT116 cells treated or not daunorubicin. The NDR viewpoint track is shown. (D) High-resolution 4C experiment carried out in HCT116 p53-/- cells treated or not with daunorubicin using DpnII as first cutter. The NDR viewpoint track is shown. (DOC) [file pone.0163885.s004.doc]

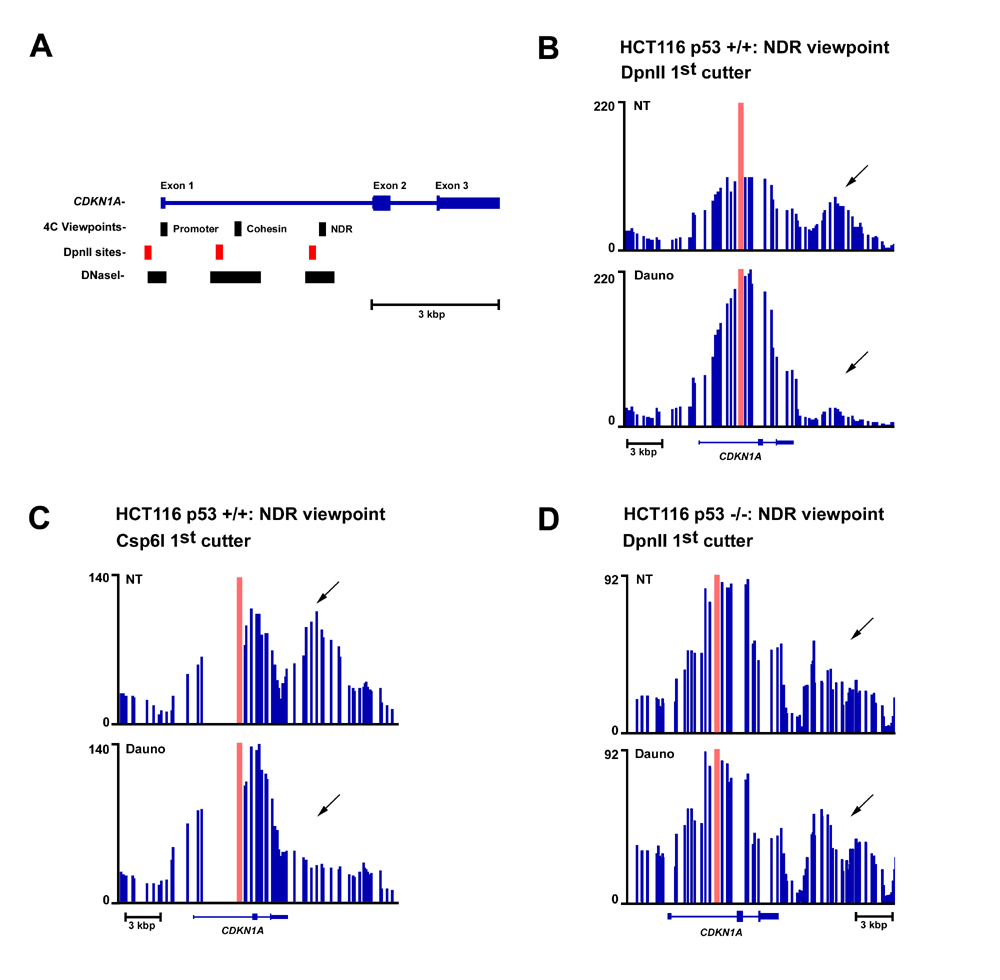


**Fig. S4**

**Figure S4. The 3D spatial organization of the *CDKN1A* locus is remodeled in response to stress.**

(**A**) Representation of the *CDKN1A* gene with the promoter, the cohesin, and the NDR 4C viewpoints. 4C DpnII restriction sites are shown in red. The ENCODE DNaseI tracks (data from the University of Washington ENCODE group on behalf of the ENCODE Analysis Working Group) adapted from the UCSC genome browser is also shown. (**B**) Biological and technical replicate of the high-resolution 4C experiment carried out in HCT116 cells treated or not with daunorubicin using DpnII as first cutter. The NDR viewpoint track is shown. (**C**) High-resolution 4C experiment performed using Csp6I as first cutter (instead of DpnII) in HCT116 cells treated or not daunorubicin. The NDR viewpoint track is shown. (**D**) High-resolution 4C experiment carried out in HCT116 p53-/- cells treated or not with daunorubicin using DpnII as first cutter. The NDR viewpoint track is shown.
