# Supplementary material for: High-Resolution 4C Reveals Rapid p53-Dependent Chromatin Reorganization of the CDKN1A Locus in Response to Stress
Source: PLoS One. 2016 Oct 14;11(10):e0163885. doi: 10.1371/journal.pone.0163885 (PMC5065170; doi:10.1371/journal.pone.0163885)
Supplement: S5 Fig — 4C high-resolution interaction map of the CDKN1A locus. HCT116 p53+/+ cells were treated (Dauno) or not (NT) with daunorubicin. The 4C tracks for the cohesin site and the interacting region are presented with their respective viewpoints in red. Rad21 ChIP-seq tracks are also shown. P53 binding sites are shown in green in the gene track. (DOC) [file pone.0163885.s005.doc]

**Fig. S5**


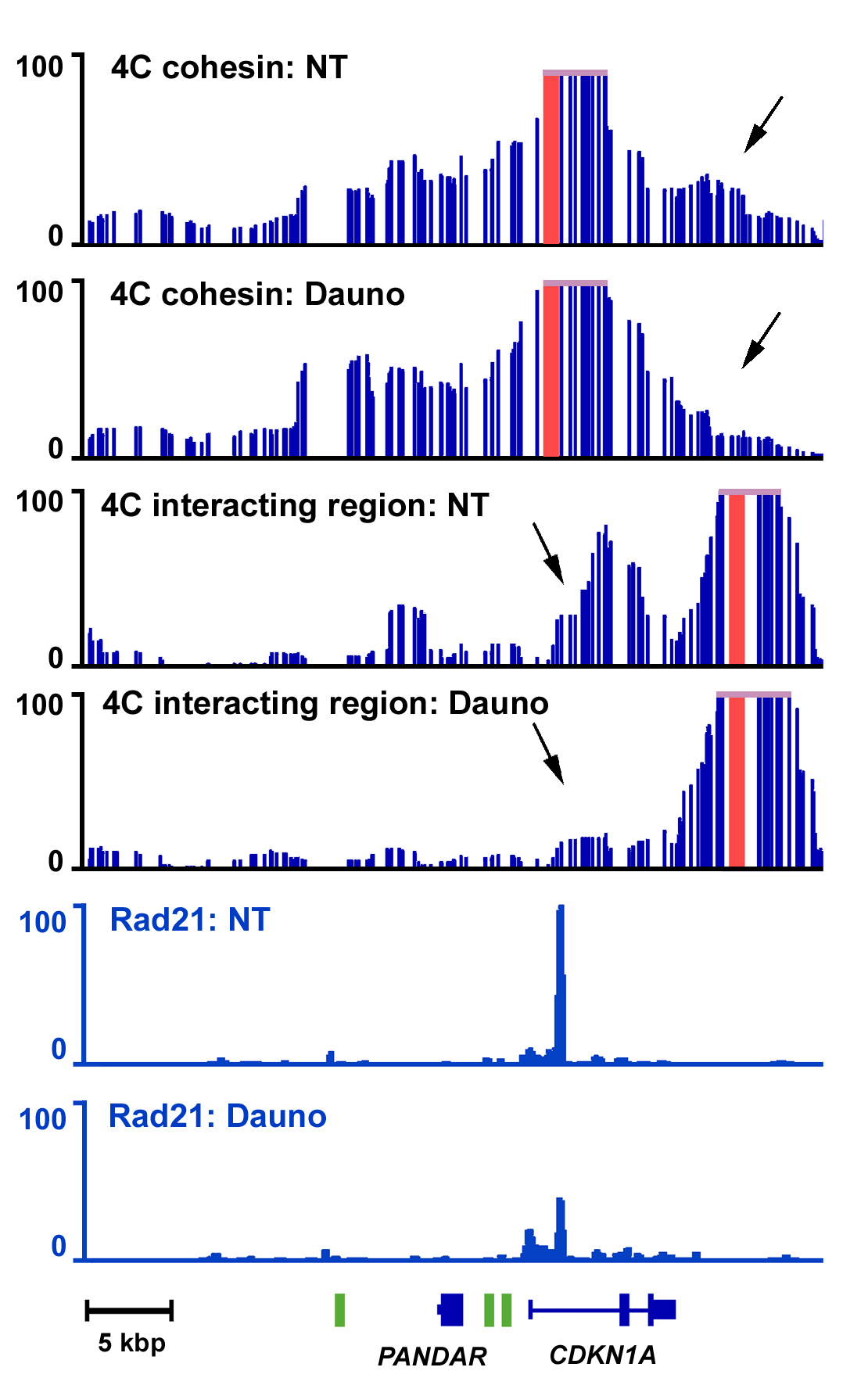


**Figure S5. The *CDKN1A* cohesin site is part of the interaction between the NDR and the downstream interacting region.**

4C high-resolution interaction map of the *CDKN1A* locus. HCT116 p53^+/+^ cells were treated (Dauno) or not (NT) with daunorubicin. The 4C tracks for the cohesin site and the interacting region are presented with their respective viewpoints in red. Rad21 ChIP-seq tracks are also shown. P53 binding sites are shown in green in the gene track.
