# Supplementary material for: High-Resolution 4C Reveals Rapid p53-Dependent Chromatin Reorganization of the CDKN1A Locus in Response to Stress
Source: PLoS One. 2016 Oct 14;11(10):e0163885. doi: 10.1371/journal.pone.0163885 (PMC5065170; doi:10.1371/journal.pone.0163885)
Supplement: S6 Fig — (A) Luciferase reporter assay to test if the internal promoter activity is affected by stress. (B) Luciferase reporter assay to test if the activity of the internal promoter is p53-dependent. (C) PCR products obtained with primers amplifying the 5’RACE / p21 exon 2 junction using gDNA as template or cDNA from daunorubicin treated HCT116 cell. (DOC) [file pone.0163885.s006.doc]

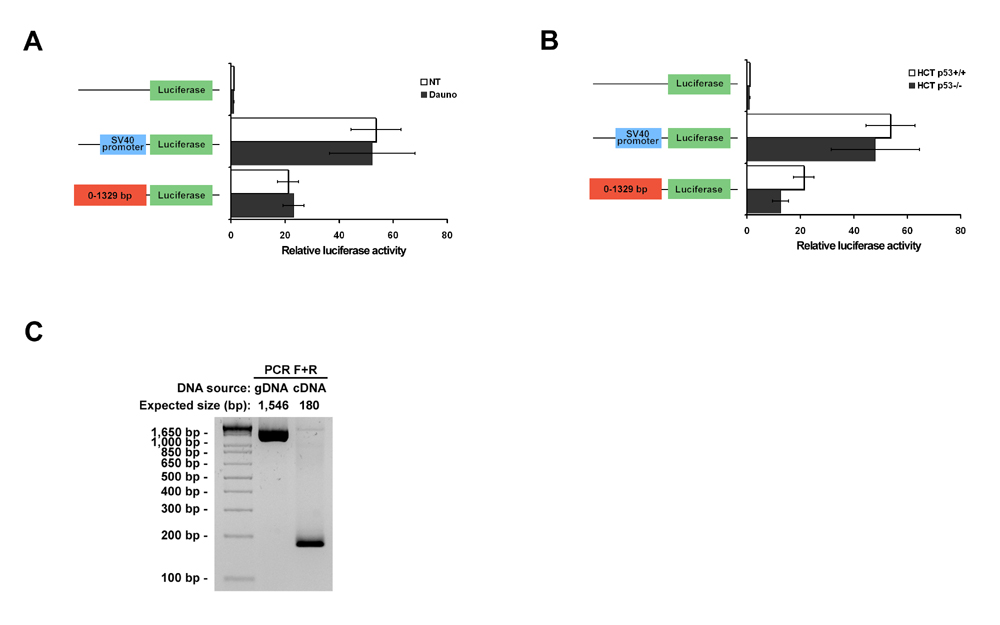


**Fig. S6**

**Figure S6. The *CDKN1A* NDR is an internal alternative promoter.**

(**A**) Luciferase reporter assay to test if the internal promoter activity is affected by stress. (**B**) Luciferase reporter assay to test if the activity of the internal promoter is p53-dependent. (**C**) PCR products obtained with primers amplifying the 5’RACE / p21 exon 2 junction using gDNA as template or cDNA from daunorubicin treated HCT116 cell.
