# Supplementary material for: High-Resolution 4C Reveals Rapid p53-Dependent Chromatin Reorganization of the CDKN1A Locus in Response to Stress
Source: PLoS One. 2016 Oct 14;11(10):e0163885. doi: 10.1371/journal.pone.0163885 (PMC5065170; doi:10.1371/journal.pone.0163885)
Supplement: S7 Fig — (A) 4C track of the NDR viewpoint obtained in non-treated HCT116 cells aligned to the ENCODE DNaseI track (data from the University of Washington ENCODE group on behalf of the ENCODE Analysis Working Group). Adapted from the UCSC genome browser. (B) FAIRE experiment carried out in non-treated HCT116 cells. Primer sets used were tailing the DNaseI peak located at chr6:36,660,086–36,660,295 strand (+) hg19. (C) Luciferase reporter assay to test the distal enhancer activity of the interacting region regulatory element. The signal is normalized to the Internal promoter/Luciferase construction. (D) Luciferase reporter assay to test the proximal enhancer activity of the interacting region regulatory element. The signal is normalized to the Control DNA1/Internal promoter/Luciferase construction. (E) Luciferase reporter assay to test the promoter activity of the interacting region regulatory element. The signal is normalized to the Control DNA1/Luciferase construction. (F) Luciferase reporter assay to test if the interacting region regulatory element represses SV40 enhancer activity. The signal is normalized to the SV40 promoter/Luciferase construction. (DOC) [file pone.0163885.s007.doc]

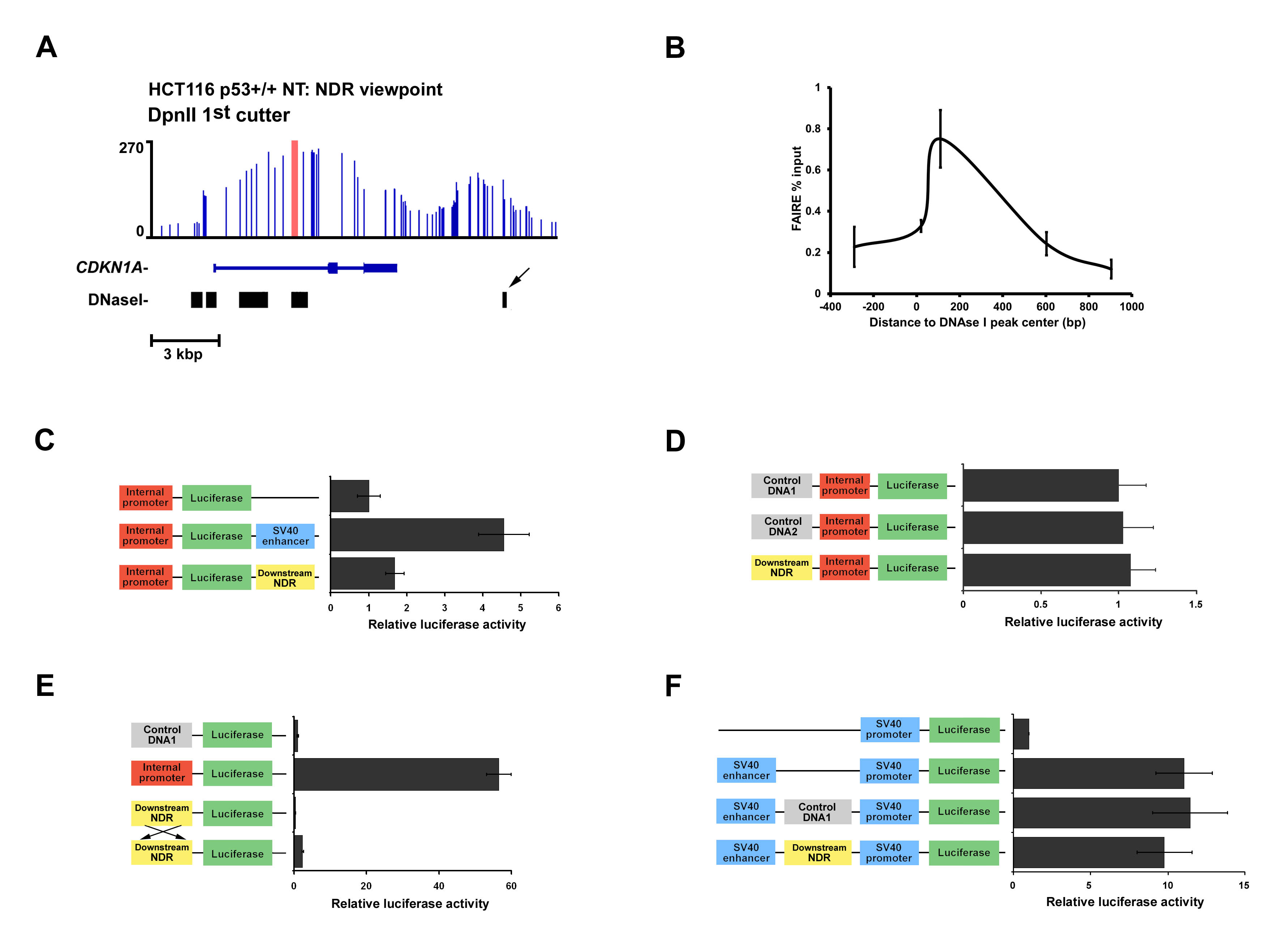


**Fig. S7**

**Figure S7. The *CDKN1A* downstream interacting region is a repressor element.**

(**A**) 4C track of the NDR viewpoint obtained in non-treated HCT116 cells aligned to the ENCODE DNaseI track (data from the University of Washington ENCODE group on behalf of the ENCODE Analysis Working Group). Adapted from the UCSC genome browser. (**B**) FAIRE experiment carried out in non-treated HCT116 cells. Primer sets used were tailing the DNaseI peak located at chr6:36,660,086-36,660,295 strand (+) hg19. (**C**) Luciferase reporter assay to test the distal enhancer activity of the interacting region regulatory element. The signal is normalized to the Internal promoter/Luciferase construction. (**D**) Luciferase reporter assay to test the proximal enhancer activity of the interacting region regulatory element. The signal is normalized to the Control DNA1/Internal promoter/Luciferase construction. (**E**) Luciferase reporter assay to test the promoter activity of the interacting region regulatory element. The signal is normalized to the Control DNA1/Luciferase construction. (**F**) Luciferase reporter assay to test if the interacting region regulatory element represses SV40 enhancer activity. The signal is normalized to the SV40 promoter/Luciferase construction.
