# Supplementary material for: High-Resolution 4C Reveals Rapid p53-Dependent Chromatin Reorganization of the CDKN1A Locus in Response to Stress
Source: PLoS One. 2016 Oct 14;11(10):e0163885. doi: 10.1371/journal.pone.0163885 (PMC5065170; doi:10.1371/journal.pone.0163885)
Supplement: S8 Fig — Venn diagram of the intersection of p53 binding sites and Rad21 binding sites in HCT116 p53+/+. (DOC) [file pone.0163885.s008.doc]

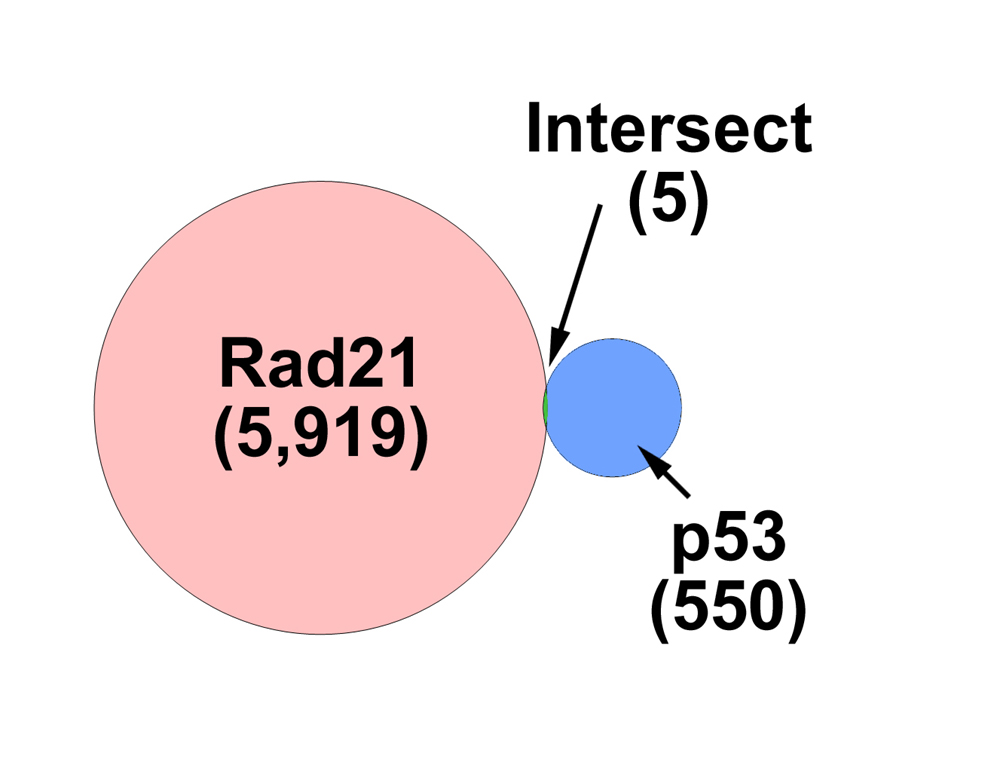


**Fig. S8**

**Figure S8. p53 does not colocalize with Rad21.**

Venn diagram of the intersection of p53 binding sites and Rad21 binding sites in HCT116 p53+/+.
