# Supplementary material for: High-Resolution 4C Reveals Rapid p53-Dependent Chromatin Reorganization of the CDKN1A Locus in Response to Stress
Source: PLoS One. 2016 Oct 14;11(10):e0163885. doi: 10.1371/journal.pone.0163885 (PMC5065170; doi:10.1371/journal.pone.0163885)
Supplement: S1 File — (DOC) [file pone.0163885.s009.doc]

**Supplementary biofinformatic procedures**

**RNA-seq data analysis**

We used the RNA-seq pipeline version 1.4 developed by MUGQIC (https://bitbucket.org/mugqic/mugqic_pipelines) to analyze our data. Briefly, the raw reads were trimmed using Trimmomatic 0.32 [1] to remove from the 3’-end the bases with a Phred quality score below 30, and remove the trimmed reads shorter than 32 bp. The trimmed reads were then aligned onto the human reference genome hg19 using TopHat 2.0.11 [2] and Bowtie 2.2.2 [3] with default parameters except --library-type fr-firststrand -G transcripts_ensembl.gtf (GRCh37.68), then MarkDuplicates from Picard 1.118 (ref) was applied. Finally FPKM values were calculated for each gene using Cufflinks 2.1.1 [4] with default parameters except -G transcripts_ensembl.gtf (GRCh37.68).

**ChIP-seq data analysis**

We based the analyses of Rad21 ChIP-Seq data on the ChIP-seq pipeline version 1.4 developed by MUGQIC. Briefly the raw reads were trimmed as for RNA-Seq reads, then aligned onto hg19 using BWA 0.7.5a with default parameters, then filtered on alignment quality using Samtools 0.1.19 with the settings view -F4 -q 20 [5]. The two biological replicates per condition were then combined using makeTagDirectory from Homer 4.1 [6]. The signal density files in BedGraph were generated using Homer 4.1 and the default settings of the command makeUCSCfile [6]​. Narrow peak summits were determined using MACS2 version 2.0.10 [7] with default parameters except --fix-bimodal -f BAMPE.

**Rad21 binding fold change calculation**

For each Rad21 peak identified by ChIP-seq in non-treated cells, the reads density +/- 120 bp around Rad21 peak summits was averaged in 20 bins of 12 bp with Versatile Aggregate Profiler 1.10 (VAP, <http://lab-jacques.recherche.usherbrooke.ca/software_en/vap/home/>) using the coordinate reference groups in relative mode with one reference point. The same approach was done for the Rad21 peaks obtained in daunorubicin treated cells, but we used Rad21 peak summit coordinates obtained in non-treated cells as reference point for the binning [8]. The signal for each Rad21 site was calculated by summing up the value of the 20 bins. The log2 fold change was determined for each Rad21 site by calculating the log2 value of the signal obtained in daunorubicin treated cells divided by the signal obtained in non-treated cells. A Z-score value of the log2 fold change was then calculated for each Rad21 site.

We observed that the noise in ChIP-seq experiment is inversely correlated to the signal intensity; the noise decrease as the signal increase (Fig S1A). In order to minimize the effect of noise on our analyses, Figure 1A, 1C, and S1E were generated using the 5,919 Rad21 sites having a summit value >50 (two times higher that the summit median of the population) in either non-stressed cells or daunorubicin treated cells (red lines Fig S1A).

**Rad21 ChIP-seq Heatmap**

Figure 1A shows the average read density signal +/- 396 bp around Rad21 peak summits. Heatmaps were generated using Gene-E (<http://www.broadinstitute.org/cancer/software/GENE-E/>). The Rad21 read signal was averaged in 66 bins of 12 bp with VAP 1.10 using the coordinate reference groups in relative mode with one reference point. For ease of viewing, for each Rad21 peak obtained in non-treated cells, the maximum signal intensity was normalized to 1 by dividing the signal of each bin by the value of the bin having the highest signal. The signal of Rad21 peaks obtained in daunorubicin treated cells was processed similarly. However the value of each bin was divided by the value of the bin, from the corresponding peak in non-treated cells, having the highest signal.

**Correlation between Rad21 fold change and transcription fold change**

Figures 1C and S1E show Rad21 binding fold change at sites located within genes plotted against the corresponding genes FPKM log2 fold change. Only significantly expressed genes with a FPKM >1 in either non-treated or daunorubicin treated conditions and having a summit value >50 in either non-treated or daunorubicin treated conditions were kept for the analysis. The data were smoothed using a moving mean with a sliding window of 500 Rad21 sites.

**Correlation between Rad21 peak summit height and transcript FPKM**

Figures 1D and S1F show the correlation between the height of Rad21 summit located within genes and the corresponding FPKM. No filters on peak height or FPKM were applied. The data were smoothed using a moving mean with a sliding window of 10,000 Rad21 sites.

**References**

1. Bolger AM, Lohse M, Usadel B. Trimmomatic: a flexible trimmer for Illumina sequence data. Bioinformatics (Oxford, England). 2014;30(15):2114-20. PubMed PMID: 24695404.

2. Kim D, Pertea G, Trapnell C, Pimentel H, Kelley R, Salzberg SL. TopHat2: accurate alignment of transcriptomes in the presence of insertions, deletions and gene fusions. Genome biology. 2013;14(4):R36. PubMed PMID: 23618408.

3. Li H, Durbin R. Fast and accurate short read alignment with Burrows-Wheeler transform. Bioinformatics (Oxford, England). 2009;25(14):1754-60. PubMed PMID: 19451168.

4. Trapnell C, Williams BA, Pertea G, Mortazavi A, Kwan G, van Baren MJ, et al. Transcript assembly and quantification by RNA-Seq reveals unannotated transcripts and isoform switching during cell differentiation. Nature biotechnology. 2010;28(5):511-5. PubMed PMID: 20436464.

5. Li H, Handsaker B, Wysoker A, Fennell T, Ruan J, Homer N, et al. The Sequence Alignment/Map format and SAMtools. Bioinformatics (Oxford, England). 2009;25(16):2078-9. PubMed PMID: 19505943.

6. Heinz S, Benner C, Spann N, Bertolino E, Lin YC, Laslo P, et al. Simple combinations of lineage-determining transcription factors prime cis-regulatory elements required for macrophage and B cell identities. Molecular cell. 2010;38(4):576-89. PubMed PMID: 20513432.

7. Zhang Y, Liu T, Meyer CA, Eeckhoute J, Johnson DS, Bernstein BE, et al. Model-based analysis of ChIP-Seq (MACS). Genome biology. 2008;9(9):R137. PubMed PMID: 18798982.

8. Coulombe C, Poitras C, Nordell-Markovits A, Brunelle M, Lavoie MA, Robert F, et al. VAP: a versatile aggregate profiler for efficient genome-wide data representation and discovery. Nucleic acids research. 2014;42(Web Server issue):W485-93. PubMed PMID: 24753414.
