# Supplementary material for: High-Resolution 4C Reveals Rapid p53-Dependent Chromatin Reorganization of the CDKN1A Locus in Response to Stress
Source: PLoS One. 2016 Oct 14;11(10):e0163885. doi: 10.1371/journal.pone.0163885 (PMC5065170; doi:10.1371/journal.pone.0163885)
Supplement: S1 Table — (DOC) [file pone.0163885.s010.doc]

**Table S1. ChIP antibodies**

| **Antibody** | **Company** | **Reference** |
| --- | --- | --- |
| **CTCF** | Milipore | 07-729 |
| **H3K4me1** | Abcam | ab8895 |
| **H3K4me3** | Milipore | 07-473 |
| **H3** | Abcam | ab1791 |
| **Rad21** | abcam | ab992 |
| **Smc1** | Bethyl | A300-055A |
