# Supplementary material for: High-Resolution 4C Reveals Rapid p53-Dependent Chromatin Reorganization of the CDKN1A Locus in Response to Stress
Source: PLoS One. 2016 Oct 14;11(10):e0163885. doi: 10.1371/journal.pone.0163885 (PMC5065170; doi:10.1371/journal.pone.0163885)
Supplement: S2 Table — (DOC) [file pone.0163885.s011.doc]

**Table S2. ChIP qPCR primers**

| ***CDKN1A* cohesin site** | | |
| --- | --- | --- |
| **Distance to cohesin site (bp)** | **Forward primer** | **Reverse primer** |
| **-1533** | TGCGTTCACAGGTGTTTCTG | CACATCCCGACCCTCGTCAC |
| **-1209** | AGCGGAGTGGAGTAAGTTCG | ACAGTGGCGTAAAGGACCTG |
| **-809** | CTGTAGGGGTCGGGGAGT | CGGTGGGAAAGAGGTAGAGG |
| **-708** | AAGGGCAAGGGTCTCCTCTA | CCACCCCTCAAAGACATGAA |
| **-359** | GGAAAAGCATCTTGGAGCTG | TAGACGGGAGCAACGAAAAC |
| **-311** | CTCTCCTTGCCTCCCTCCAC | TATCTCCACGCCCAAAGCAC |
| **0** | AGCCGGAGTGGAAGCAGT | AGTGATGAGTCAGTTTCCTGCAAG |
| **241** | TATGCCATTACCACCCC | GGCCTTCCTGCCTTAGA |
| **682** | ACCCAAGAGTGCCCATCTGA | CTGCCTCCCGAGACCAACT |
| **1512** | TTGGGCTCAAATGATTCTCC | TAACCAGAGAGGCCCATCAC |
| **2120** | ATGTGTCCCGGGCTTCCT | CGACCTTGGCAGCAACTG |
| **2820** | TGCCCAGGGTCACCTAGTAA | CAGCAAGGCAGACAGAACAGA |

| **Cohesin site** | **Forward primer** | **Reverse primer** |
| --- | --- | --- |
| ***FDXR*** | CATCAGACACTGACCGAGGA | GTTGGAGCCATTTGCAGAGT |
| ***TP53i3*** | CTCCTGCCTGGGAAGTCCT | CTCTTCGGCGTTGTCCTG |
| ***GDF15*** | GCAACGGGGACCACTGTC | CCAGCCCAGGTCTTCCAG |
