# Supplementary material for: High-Resolution 4C Reveals Rapid p53-Dependent Chromatin Reorganization of the CDKN1A Locus in Response to Stress
Source: PLoS One. 2016 Oct 14;11(10):e0163885. doi: 10.1371/journal.pone.0163885 (PMC5065170; doi:10.1371/journal.pone.0163885)
Supplement: S3 Table — (DOC) [file pone.0163885.s012.doc]

**Table S3. RT-qPCR primers**

| **mRNA** | **Forward primer** | **Reverse primer** |
| --- | --- | --- |
| **p21** | GGAGACTCTCAGGGTCGAAA | GGATTAGGGCTTCCTCTTGG |
| **p21C** | ATCCAGTTGCTGCCAAGGTC | CCATTAGCGCATCACAGTCG |
| **36B4** | CGACCTGGAAGTCCAACTAC | ATCTGCTGCATCTGCTTG |
