# Supplementary material for: High-Resolution 4C Reveals Rapid p53-Dependent Chromatin Reorganization of the CDKN1A Locus in Response to Stress
Source: PLoS One. 2016 Oct 14;11(10):e0163885. doi: 10.1371/journal.pone.0163885 (PMC5065170; doi:10.1371/journal.pone.0163885)
Supplement: S4 Table — (DOC) [file pone.0163885.s013.doc]

**Table S4. FAIRE qPCR primers**

| ***CDKN1A* cohesin site** | | |
| --- | --- | --- |
| **Identical to the ChIP primers** | | |
| ***CDKN1A* downstream NDR** | | |
| **Distance to DNaseI site (bp)** | **Forward primer** | **Reverse primer** |
| **-289** | AACTGGGGCTTAAACCTGCT | GAATTTCTTCCACCCCATGA |
| **20** | GGATGTCCACGCTGATGACT | AGAGGTGGGTCCCTGCAA |
| **110** | CTTGCAGGGACCCACCTC | TGAAGCAAGCTCCCTGACC |
| **603** | CCTAGCGCTTGGTGTGTCC | CTTGCCTTCCCCATGTCC |
| **904** | GGTCCTGGACTTGTTGCATT | AGGGAGACCCATGAGGAGTT |
