# Supplementary material for: High-Resolution 4C Reveals Rapid p53-Dependent Chromatin Reorganization of the CDKN1A Locus in Response to Stress
Source: PLoS One. 2016 Oct 14;11(10):e0163885. doi: 10.1371/journal.pone.0163885 (PMC5065170; doi:10.1371/journal.pone.0163885)
Supplement: S5 Table — (DOC) [file pone.0163885.s014.doc]

**Table S5. 4C primers**

| **DpnII first cutter*** | | |
| --- | --- | --- |
| ***CDKN1A***  **Promoter** | **Reading** | AATGATACGGCGACCACCGAACACTCTTTCCCTACACGACGCTCTTCCGATCT**XX**CCAATGCAGCTGAAAAGATC |
|  | **Reverse** | CAAGCAGAAGACGGCATACGACTTTGGCTGCGGTTGGCT |
| ***CDKN1A* NDR** | **Reading** | AATGATACGGCGACCACCGAACACTCTTTCCCTACACGACGCTCTTCCGATCT**XX**ACTGTGGGCTCCACTGAT |
|  | **Reverse** | CAAGCAGAAGACGGCATACGATACAGCTAAAAAGGCCAAAG |
| ***CDKN1A***  **Downstream NDR** | **Reading** | AATGATACGGCGACCACCGAACACTCTTTCCCTACACGACGCTCTTCCGATCT**XX**CTTCACTGGTGAGGGATC |
|  | **Reverse** | CAAGCAGAAGACGGCATACGAGGGCAACACTGACTTACACT |
| ***FDXR*** | **Reading** | AATGATACGGCGACCACCGAACACTCTTTCCCTACACGACGCTCTTCCGATCT**XX**CCTCTCAGAGCTTGTGATC |
|  | **Reverse** | CAAGCAGAAGACGGCATACGACGAGTGAAGACAGTGTGG |
| **Csp6I first cutter*** | | |
| ***CDKN1A* NDR** | **Reading** | AATGATACGGCGACCACCGAACACTCTTTCCCTACACGACGCTCTTCCGATCT**XX**ATAAAGGGGTCACCCAGTAC |
|  | **Reverse** | CAAGCAGAAGACGGCATACGAATGAGGACTCAGCAGAGCT |

*****XX=tag, CG non treated, GC daunorubicin
