# Supplementary material for: High-Resolution 4C Reveals Rapid p53-Dependent Chromatin Reorganization of the CDKN1A Locus in Response to Stress
Source: PLoS One. 2016 Oct 14;11(10):e0163885. doi: 10.1371/journal.pone.0163885 (PMC5065170; doi:10.1371/journal.pone.0163885)
Supplement: S6 Table — (DOC) [file pone.0163885.s015.doc]

**Table S6. Luciferase reporter assay genomic sequences**

| **Internal promoter sequences** | |
| --- | --- |
| **0-325** | chr6(+):36,649,572-36,649,896 (hg19) |
| **0-589** | chr6(+):36,649,572-36,650,160 (hg19) |
| **0-989** | chr6(+):36,649,572-36,650,560 (hg19) |
| **0-1327** | chr6(+):36,649,572-36,650,898 (hg19) |
| **529-989**  **(Internal promoter)** | chr6(+):36,650,101-36,650,560 (hg19) |
| **Interacting region sequence** | |
| chr6(+):36,660,065-36,660,398 (hg19) | |
| **Control DNA sequences** | |
| **Control DNA1** | chr6(+):36,675,422-36,675,605 (hg19) |
| **Control DNA2** | chr6(+):36,650,561-36,650,898 (hg19) |
