# Supplementary material for: High-Resolution 4C Reveals Rapid p53-Dependent Chromatin Reorganization of the CDKN1A Locus in Response to Stress
Source: PLoS One. 2016 Oct 14;11(10):e0163885. doi: 10.1371/journal.pone.0163885 (PMC5065170; doi:10.1371/journal.pone.0163885)
Supplement: S7 Table — (DOC) [file pone.0163885.s016.doc]

**Table S7. 5’RACE primers**

| **mRNA** | **GSP1** | **GSP2** | **GSP3** |
| --- | --- | --- | --- |
| **p21** | GCACAAGGGTACAAGACAGT | ACACGACGCTCTTCCGATCT | GTCGAAGTTCCATCGCTCAC |
| **Int. prom.**  **primer set #1** | CACATGCCACATCTGCTCCA | CACATGCCACATCTGCTCCA | TGCTTGTGATCAGTGGAGCC |
| **Int. prom.**  **primer set #2** | CCTGCCTGCTCT | CCTGCCTGCTCTGGCTTG | TTGACTCCAGGGCTGAGTGA |
