# Supplementary material for: High-Resolution 4C Reveals Rapid p53-Dependent Chromatin Reorganization of the CDKN1A Locus in Response to Stress
Source: PLoS One. 2016 Oct 14;11(10):e0163885. doi: 10.1371/journal.pone.0163885 (PMC5065170; doi:10.1371/journal.pone.0163885)
Supplement: S8 Table — (DOC) [file pone.0163885.s017.doc]

**Table S8**. Coordinates of cohesin binding sites with a Z-score <-2 or >2.

| **Z-score < -2** | | | | | |
| --- | --- | --- | --- | --- | --- |
| **Chr** | **Summit coord** | **Summit Log2(DAU/NT)** | **Z-score** | **Associated gene** | **p53 regulated** |
| chr6 | 36648373 | -1,40 | -6,98 | CDKN1A | Yes |
| chr7 | 44347112 | -1,08 | -5,33 | CAMK2B |  |
| chr17 | 72861160 | -0,98 | -4,83 | FDXR | Yes |
| chr4 | 154154074 | -0,89 | -4,33 | TRIM2 |  |
| chr4 | 1026471 | -0,84 | -4,08 |  |  |
| chr22 | 24643697 | -0,82 | -4,01 |  |  |
| chr7 | 100063616 | -0,81 | -3,93 | TSC22D4 |  |
| chr6 | 154936960 | -0,79 | -3,84 |  |  |
| chr8 | 27472380 | -0,77 | -3,72 | CLU |  |
| chr7 | 5527630 | -0,75 | -3,62 | FBXL18 |  |
| chr1 | 45265534 | -0,68 | -3,29 |  |  |
| chr8 | 95003785 | -0,68 | -3,25 |  |  |
| chr16 | 22217495 | -0,67 | -3,21 |  |  |
| chr8 | 95274609 | -0,67 | -3,20 |  |  |
| chr19 | 18499397 | -0,66 | -3,17 | GDF15 | Yes |
| chr12 | 123237229 | -0,66 | -3,15 |  |  |
| chr17 | 29860835 | -0,65 | -3,12 | RAB11FIP4 |  |
| chr17 | 14206066 | -0,65 | -3,10 | HS3ST3B1 |  |
| chr1 | 201278816 | -0,64 | -3,08 | PKP1 |  |
| chr2 | 10237692 | -0,63 | -3,00 |  |  |
| chr17 | 16764717 | -0,62 | -2,97 |  |  |
| chr19 | 42392000 | -0,62 | -2,97 | ARHGEF1 |  |
| chr16 | 57509965 | -0,62 | -2,94 | DOK4 |  |
| chr17 | 46680087 | -0,62 | -2,93 | HOXB6; HOXB3; HOXB-AS3 |  |
| chr17 | 76311661 | -0,62 | -2,93 |  |  |
| chr20 | 5107246 | -0,61 | -2,93 | PCNA | Yes |
| chr14 | 77413647 | -0,61 | -2,91 |  |  |
| chr5 | 175843666 | -0,60 | -2,85 |  |  |
| chr3 | 58223434 | -0,60 | -2,84 | ABHD6 |  |
| chr10 | 123872276 | -0,60 | -2,84 | TACC2 |  |
| chr2 | 219575611 | -0,59 | -2,82 | TTLL4 |  |
| chr20 | 44882221 | -0,59 | -2,82 | CDH22 |  |
| chr12 | 54973683 | -0,59 | -2,82 | PPP1R1A |  |
| chr4 | 187647170 | -0,59 | -2,78 | FAT1 |  |
| chr7 | 61968977 | -0,58 | -2,76 |  |  |
| chr1 | 154377747 | -0,58 | -2,76 | IL6R; RP11-350G8.5 |  |
| chr17 | 15848089 | -0,58 | -2,73 |  |  |
| chr12 | 13356068 | -0,57 | -2,72 | EMP1 |  |
| chr17 | 70654100 | -0,57 | -2,71 | SLC39A11 |  |
| chr8 | 22876901 | -0,57 | -2,69 | RHOBTB2 |  |
| chr2 | 24307358 | -0,56 | -2,63 | TP53I3; AC008073.6 | Yes |
| chr6 | 144537503 | -0,56 | -2,63 |  |  |
| chr22 | 47135188 | -0,55 | -2,61 |  |  |
| chr4 | 155548077 | -0,55 | -2,60 |  |  |
| chr22 | 39162308 | -0,55 | -2,60 | SUN2 |  |
| chr17 | 79486522 | -0,55 | -2,59 | ACTG1; RP13-766D20.2 |  |
| chr15 | 89787075 | -0,55 | -2,58 |  |  |
| chr4 | 99916500 | -0,54 | -2,57 |  |  |
| chr22 | 37714650 | -0,54 | -2,56 |  |  |
| chr16 | 68772269 | -0,54 | -2,55 | CDH1 |  |
| chr6 | 44119761 | -0,54 | -2,55 | TMEM63B |  |
| chr17 | 79283538 | -0,54 | -2,54 |  |  |
| chr19 | 35738857 | -0,54 | -2,54 |  |  |
| chr2 | 192109942 | -0,54 | -2,52 | MYO1B; AC092614.2 |  |
| chr16 | 88450345 | -0,53 | -2,51 |  |  |
| chr11 | 65184009 | -0,53 | -2,51 |  |  |
| chr4 | 169506828 | -0,53 | -2,50 | PALLD |  |
| chr14 | 101550285 | -0,53 | -2,48 |  |  |
| chr1 | 121485194 | -0,53 | -2,47 |  |  |
| chr17 | 38519013 | -0,52 | -2,46 | GJD3; CTD-2267D19.3 |  |
| chr7 | 148787941 | -0,52 | -2,46 |  |  |
| chr12 | 54762622 | -0,52 | -2,43 | RP11-753H16.5; RP11-753H16.3 |  |
| chr12 | 95611344 | -0,52 | -2,42 |  |  |
| chr2 | 112655662 | -0,52 | -2,42 |  |  |
| chr15 | 89954924 | -0,51 | -2,41 |  |  |
| chr20 | 33543732 | -0,51 | -2,41 |  |  |
| chr20 | 33061249 | -0,51 | -2,40 | ITCH |  |
| chr17 | 48157226 | -0,51 | -2,39 | ITGA3 |  |
| chr18 | 33876775 | -0,51 | -2,39 |  |  |
| chr5 | 72747267 | -0,51 | -2,39 |  |  |
| chr5 | 131862433 | -0,51 | -2,38 |  |  |
| chr19 | 42462112 | -0,51 | -2,36 | RABAC1 |  |
| chr20 | 44937428 | -0,50 | -2,35 |  |  |
| chr17 | 77890083 | -0,50 | -2,33 | RP11-353N14.5 |  |
| chr17 | 16894899 | -0,50 | -2,33 |  |  |
| chr17 | 71152517 | -0,50 | -2,32 |  |  |
| chr3 | 127409903 | -0,50 | -2,32 | MGLL |  |
| chr3 | 48545233 | -0,49 | -2,31 |  |  |
| chr5 | 10284298 | -0,49 | -2,31 | CMBL |  |
| chr16 | 71392556 | -0,49 | -2,30 |  |  |
| chr5 | 68855921 | -0,49 | -2,29 | GUSBP3 |  |
| chr1 | 10590529 | -0,49 | -2,29 | PEX14 |  |
| chr1 | 156100268 | -0,48 | -2,26 | LMNA |  |
| chr22 | 39323475 | -0,48 | -2,23 |  |  |
| chr3 | 129047704 | -0,48 | -2,23 |  |  |
| chrX | 46763292 | -0,48 | -2,22 |  |  |
| chr12 | 52672924 | -0,48 | -2,21 | AC021066.1; RP11-845M18.7 |  |
| chr1 | 228566503 | -0,47 | -2,20 | OBSCN |  |
| chr17 | 73852149 | -0,47 | -2,16 | WBP2 |  |
| chr11 | 64948473 | -0,47 | -2,16 | CAPN1; AP003068.23 |  |
| chr21 | 43198553 | -0,46 | -2,15 |  |  |
| chr2 | 97523982 | -0,46 | -2,15 |  |  |
| chr1 | 950954 | -0,46 | -2,15 |  |  |
| chr1 | 48559552 | -0,46 | -2,15 |  |  |
| chr5 | 171615840 | -0,46 | -2,14 |  |  |
| chr12 | 132990726 | -0,46 | -2,14 |  |  |
| chr12 | 131601082 | -0,46 | -2,14 | GPR133 |  |
| chr19 | 42795226 | -0,46 | -2,13 | CIC |  |
| chr14 | 69255780 | -0,46 | -2,13 | ZFP36L1 |  |
| chr1 | 114447864 | -0,46 | -2,13 | DCLRE1B |  |
| chr1 | 183155427 | -0,46 | -2,11 | LAMC2 |  |
| chr22 | 51001305 | -0,46 | -2,11 | SYCE3 |  |
| chr1 | 224828916 | -0,45 | -2,10 | CNIH3 |  |
| chr20 | 25842394 | -0,45 | -2,10 | FAM182B |  |
| chr5 | 132165791 | -0,45 | -2,09 | SHROOM1 |  |
| chr17 | 42161024 | -0,45 | -2,09 | HDAC5 |  |
| chr22 | 23745065 | -0,45 | -2,08 |  |  |
| chr19 | 18390391 | -0,45 | -2,08 |  |  |
| chr18 | 77929293 | -0,45 | -2,08 | PARD6G |  |
| chr11 | 64764425 | -0,45 | -2,08 | BATF2 |  |
| chr2 | 218899628 | -0,45 | -2,07 |  |  |
| chr17 | 34091258 | -0,45 | -2,06 | MMP28; C17orf50 |  |
| chr7 | 5526690 | -0,44 | -2,05 | FBXL18 |  |
| chr19 | 19286008 | -0,44 | -2,05 | MEF2BNB-MEF2B |  |
| chr8 | 143690599 | -0,44 | -2,04 |  |  |
| chr20 | 61804245 | -0,44 | -2,03 | RP5-963E22.4 |  |
| chr16 | 56677490 | -0,44 | -2,03 |  |  |
| chr16 | 4986511 | -0,44 | -2,03 | PPL |  |
| chr10 | 112431992 | -0,44 | -2,02 | RBM20 |  |
| chr15 | 40971507 | -0,44 | -2,02 |  |  |
| chr17 | 42193178 | -0,44 | -2,02 | HDAC5 |  |
| chr17 | 57297880 | -0,44 | -2,02 | GDPD1 |  |
| chr15 | 66544891 | -0,44 | -2,02 | MEGF11 |  |
| chr17 | 17291561 | -0,44 | -2,02 |  |  |
| chr16 | 30965166 | -0,44 | -2,01 | ORAI3; AC135048.13 |  |
| chr17 | 43226712 | -0,44 | -2,01 | HEXIM1 |  |
| chr7 | 102090914 | -0,44 | -2,01 | ORAI2 |  |
| chr8 | 142639709 | -0,44 | -2,01 |  |  |
| chr16 | 1463820 | -0,44 | -2,01 | UNKL |  |
| chr8 | 23104418 | -0,44 | -2,00 | CHMP7 |  |
| **Z-score > 2** | | | | | |
| **Chr** | **Summit coord** | **Summit Log2(DAU/NT)** | **Z-score** | **Associated gene** | **p53 regulated** |
| chr22 | 17600581 | 0,83 | 4,5 | CECR6 |  |
| chr1 | 248100398 | 0,82 | 4,44 | RP11-438H8.8 |  |
| chr16 | 81232472 | 0,78 | 4,26 | PKD1L2 |  |
| chr10 | 123422638 | 0,77 | 4,2 |  |  |
| chr10 | 102098373 | 0,75 | 4,11 | RP11-34D15.2 |  |
| chr2 | 70875189 | 0,75 | 4,1 | ADD2 |  |
| chr15 | 79888578 | 0,73 | 3,99 |  |  |
| chrM | 13009 | 0,72 | 3,92 |  |  |
| chr22 | 42337443 | 0,69 | 3,8 | CENPM |  |
| chr3 | 128309966 | 0,65 | 3,58 |  |  |
| chr10 | 90488329 | 0,65 | 3,58 | LIPK; KRT8P38 |  |
| chr16 | 88764695 | 0,63 | 3,49 | RNF166; RP5-1142A6.5 |  |
| chr19 | 32778917 | 0,62 | 3,4 |  |  |
| chr8 | 140418189 | 0,61 | 3,37 |  |  |
| chr7 | 28060104 | 0,61 | 3,36 | JAZF1 |  |
| chr1 | 30266065 | 0,6 | 3,33 |  |  |
| chr4 | 15958054 | 0,6 | 3,32 |  |  |
| chr17 | 56525011 | 0,59 | 3,26 | HSF5 |  |
| chr1 | 7599950 | 0,57 | 3,18 | CAMTA1 |  |
| chr1 | 84326290 | 0,56 | 3,12 | RP11-475O6.1 |  |
| chr16 | 27709755 | 0,53 | 2,96 | KIAA0556; CTD-2049O4.1 |  |
| chr1 | 157961946 | 0,53 | 2,96 |  |  |
| chr17 | 71779379 | 0,53 | 2,95 | LINC00469 |  |
| chr12 | 54754803 | 0,53 | 2,95 | RP11-753H16.5; RP11-753H16.3 |  |
| chr17 | 8689296 | 0,52 | 2,92 |  |  |
| chr7 | 98062801 | 0,52 | 2,89 |  |  |
| chr4 | 1914959 | 0,51 | 2,85 | WHSC1 |  |
| chr17 | 71185873 | 0,51 | 2,84 | RP11-143K11.5 |  |
| chr12 | 46466107 | 0,5 | 2,8 |  |  |
| chr21 | 16855656 | 0,5 | 2,79 |  |  |
| chr10 | 47081132 | 0,49 | 2,78 | RP11-314P12.2 |  |
| chr20 | 32255863 | 0,5 | 2,78 | NECAB3; ACTL10 |  |
| chr9 | 36991704 | 0,49 | 2,73 | PAX5 |  |
| chr20 | 45149831 | 0,48 | 2,72 |  |  |
| chr17 | 62935176 | 0,48 | 2,7 | RP11-927P21.6 |  |
| chr16 | 85381038 | 0,48 | 2,68 |  |  |
| chr3 | 195502016 | 0,47 | 2,67 | MUC4 |  |
| chr22 | 49944790 | 0,47 | 2,66 | C22orf34; RP1-29C18.10 |  |
| chr6 | 143658921 | 0,47 | 2,64 | AIG1 |  |
| chr9 | 131410739 | 0,47 | 2,64 | WDR34 |  |
| chr7 | 2249274 | 0,47 | 2,64 | MAD1L1 | Yes |
| chr20 | 42846172 | 0,47 | 2,63 | RP5-995J12.2 |  |
| chr10 | 128437843 | 0,47 | 2,63 |  |  |
| chr2 | 240028182 | 0,46 | 2,61 | HDAC4 |  |
| chr17 | 66168758 | 0,46 | 2,59 |  |  |
| chr8 | 10131518 | 0,45 | 2,56 | MSRA |  |
| chr10 | 102577427 | 0,45 | 2,53 | PAX2 |  |
| chr14 | 70070484 | 0,44 | 2,52 |  |  |
| chr20 | 2736837 | 0,44 | 2,52 | EBF4 |  |
| chr8 | 97345587 | 0,45 | 2,52 | PTDSS1 |  |
| chr17 | 76570247 | 0,45 | 2,52 | DNAH17 |  |
| chr11 | 72948094 | 0,44 | 2,51 |  |  |
| chr12 | 2166576 | 0,44 | 2,5 | CACNA1C |  |
| chr3 | 61550429 | 0,44 | 2,49 | PTPRG |  |
| chr20 | 18257547 | 0,44 | 2,49 |  |  |
| chr15 | 59821255 | 0,44 | 2,49 |  |  |
| chr20 | 61300044 | 0,44 | 2,49 | SLCO4A1 |  |
| chr19 | 35634002 | 0,44 | 2,48 | FXYD1 |  |
| chr8 | 132041185 | 0,43 | 2,46 | ADCY8 |  |
| chr1 | 200948894 | 0,43 | 2,45 | KIF21B |  |
| chr17 | 15074942 | 0,43 | 2,44 |  |  |
| chr2 | 239693830 | 0,43 | 2,43 |  |  |
| chr11 | 1846369 | 0,42 | 2,41 |  |  |
| chr16 | 49384597 | 0,42 | 2,39 |  |  |
| chr17 | 49449188 | 0,42 | 2,38 |  |  |
| chr2 | 1554644 | 0,41 | 2,37 | AC144450.1 |  |
| chr2 | 19946573 | 0,42 | 2,37 |  |  |
| chr7 | 66836989 | 0,42 | 2,37 |  |  |
| chr10 | 106340725 | 0,42 | 2,37 |  |  |
| chr17 | 18529145 | 0,41 | 2,36 |  |  |
| chr3 | 131748313 | 0,41 | 2,36 | CPNE4 |  |
| chr2 | 133026220 | 0,41 | 2,34 |  |  |
| chr21 | 35802201 | 0,41 | 2,34 |  |  |
| chr15 | 86402210 | 0,41 | 2,32 |  |  |
| chr4 | 668172 | 0,4 | 2,31 | MYL5 |  |
| chr2 | 217148438 | 0,4 | 2,31 | MARCH4 |  |
| chr19 | 31160518 | 0,4 | 2,3 | ZNF536 |  |
| chr8 | 135490891 | 0,4 | 2,29 | ZFAT |  |
| chr15 | 44196205 | 0,4 | 2,28 | FRMD5 |  |
| chr3 | 37783324 | 0,4 | 2,27 | ITGA9 |  |
| chr3 | 127192254 | 0,39 | 2,26 |  |  |
| chr16 | 1814158 | 0,39 | 2,26 | MAPK8IP3 |  |
| chr20 | 58632649 | 0,39 | 2,26 | C20orf197 |  |
| chr1 | 113261486 | 0,4 | 2,26 |  |  |
| chr1 | 19977809 | 0,39 | 2,25 | NBL1 |  |
| chr2 | 84542156 | 0,39 | 2,25 |  |  |
| chr11 | 92967954 | 0,39 | 2,24 |  |  |
| chr19 | 17316260 | 0,39 | 2,24 | MYO9B |  |
| chr22 | 39939317 | 0,39 | 2,24 |  |  |
| chr10 | 73716072 | 0,39 | 2,24 |  |  |
| chr16 | 8617825 | 0,39 | 2,24 |  |  |
| chr9 | 136658302 | 0,39 | 2,23 | VAV2 |  |
| chr12 | 117135314 | 0,39 | 2,23 | RP11-497G19.1 |  |
| chr8 | 123685914 | 0,38 | 2,2 | RP11-973F15.1 |  |
| chr1 | 15354186 | 0,38 | 2,2 | KAZN |  |
| chr19 | 36551513 | 0,38 | 2,19 | WDR62 |  |
| chr7 | 99730442 | 0,38 | 2,19 | AC073842.19 |  |
| chr6 | 44235974 | 0,38 | 2,19 |  |  |
| chr17 | 45439791 | 0,38 | 2,18 | C17orf57 |  |
| chr16 | 68362669 | 0,38 | 2,18 | PRMT7 |  |
| chr20 | 31879851 | 0,38 | 2,18 | BPIFB1 |  |
| chr3 | 184521210 | 0,38 | 2,18 |  |  |
| chr11 | 117690000 | 0,38 | 2,17 | FXYD2; RP11-728F11.3 |  |
| chr2 | 28844164 | 0,38 | 2,17 | PLB1 |  |
| chr16 | 89733595 | 0,38 | 2,16 | C16orf55 |  |
| chr17 | 34982529 | 0,37 | 2,15 |  |  |
| chr6 | 161171401 | 0,37 | 2,15 | PLG |  |
| chr10 | 50189574 | 0,37 | 2,15 | WDFY4; RP11-523O18.5 |  |
| chr1 | 6101697 | 0,37 | 2,15 | KCNAB2 |  |
| chr13 | 113689404 | 0,37 | 2,14 | MCF2L |  |
| chr10 | 106065491 | 0,37 | 2,13 |  |  |
| chr6 | 39315787 | 0,37 | 2,12 | KIF6 |  |
| chr3 | 27295491 | 0,37 | 2,12 | NEK10 |  |
| chr2 | 88486776 | 0,37 | 2,11 |  |  |
| chr22 | 31957444 | 0,36 | 2,1 | SFI1 |  |
| chr19 | 8808674 | 0,36 | 2,1 | ACTL9 |  |
| chr4 | 596633 | 0,36 | 2,1 |  |  |
| chr1 | 27293583 | 0,36 | 2,1 |  |  |
| chr10 | 100022502 | 0,36 | 2,09 | LOXL4 |  |
| chr8 | 144140211 | 0,36 | 2,09 | C8orf31 |  |
| chr6 | 5215175 | 0,36 | 2,09 | LYRM4 |  |
| chr2 | 236688240 | 0,36 | 2,09 | AGAP1; AC064874.1 |  |
| chr10 | 44806625 | 0,36 | 2,08 | CXCL12 |  |
| chr1 | 22992456 | 0,36 | 2,08 |  |  |
| chr19 | 43821060 | 0,36 | 2,07 |  |  |
| chr10 | 46983840 | 0,35 | 2,06 |  |  |
| chr1 | 160651492 | 0,35 | 2,06 | CD48; RP11-404F10.2 |  |
| chr7 | 54732200 | 0,36 | 2,06 |  |  |
| chr1 | 146966981 | 0,36 | 2,06 |  |  |
| chr3 | 188672597 | 0,35 | 2,05 | TPRG1 |  |
| chr17 | 32688654 | 0,35 | 2,05 | CCL1 |  |
| chr11 | 65393628 | 0,35 | 2,05 | PCNXL3 |  |
| chr16 | 2245054 | 0,35 | 2,05 | CASKIN1 |  |
| chr2 | 114006906 | 0,35 | 2,05 | PAX8; AC016683.6 |  |
| chr10 | 118934591 | 0,35 | 2,05 |  |  |
| chr22 | 41922915 | 0,35 | 2,05 | ACO2; POLR3H |  |
| chr2 | 15044080 | 0,35 | 2,04 | AC068286.1 |  |
| chr2 | 174267325 | 0,35 | 2,03 |  |  |
| chr16 | 86907968 | 0,35 | 2,03 |  |  |
| chr10 | 124039367 | 0,34 | 2,01 | BTBD16 |  |
| chr16 | 71401466 | 0,35 | 2,01 | CALB2 |  |
| chr17 | 56394900 | 0,35 | 2,01 | BZRAP1 |  |
| chr11 | 32195133 | 0,34 | 2 | RP1-65P5.1 |  |
