# Supplementary material for: High-Resolution 4C Reveals Rapid p53-Dependent Chromatin Reorganization of the CDKN1A Locus in Response to Stress
Source: PLoS One. 2016 Oct 14;11(10):e0163885. doi: 10.1371/journal.pone.0163885 (PMC5065170; doi:10.1371/journal.pone.0163885)
Supplement: S9 Table — (DOC) [file pone.0163885.s018.doc]

**TABLE S9. 5’ RACE sequencing results**

|  | **Sequence** |
| --- | --- |
| **5’RACE p21** | ***ACACGACGCTCTTCCGATCT***GAGGTGTGAGCAGCTGCCGAAGTCAGTTCCTTGTGGAGCCGGAGCTGGGCGCGGATTCGCCGAGGCACCGAGGCACTCAGAGGAGGCGCCATGTCAGAACCGGCTGGGGATGTCCGTCAGAACCCATGCGGCAGCAAGGCCTGCCGCCGCCTCTTCGGCCCAGTGACAGCGAGCAGCTGAGCCGCGACTGTGATGCGCAATGCGCGCCCGC |
| **5’RACE internal promoter using**  **primer set #1** | ***ACACGACGCTCTTCCGATCT***TCAGGGGCAAGTCTCATATATCTTCCATCTCCTGCCCTTAAACTGGGTGGAAGTACCAAGAGCTCTCTCCACCAGCGACGAAG |
| **5’RACE internal promoter using**  **primer set #2** | ***ACACGACGCTCTTCCGATCT***TCAGGGGCAAGTCTCATATCTCTTCCATCTCCTGCCCTTTAAACTTGGTGTGAAGTTACCAAGAGCCTCCTCTCCCAACCAGCTGGGACGTGAAACTGTGGGCTCCACTGATCACAAGCAGTGGGGTGAGGTGGGGTGGAGCAGATGTGGCATGTGTCCCGGGCTTCCTGCCTCATGAGGACTCAGCAGAGCTTTCACCCCCAGAAACTGCAAGTGGGACTGTCCTAGAAATCCAGGGCCAG |

***ACACGACGCTCTTCCGATCT*** = 5’ RACE forward adapter
